# An Improved Northern Goshawk Optimization Algorithm for Mural Image Segmentation

**DOI:** 10.3390/biomimetics10060373

**Published:** 2025-06-05

**Authors:** Jianfeng Wang, Zuowen Bao, Hao Dong

**Affiliations:** 1 College of Design, Hanyang University, Ansan 15588, Republic of Korea; 15306435766@163.com; 2 College of Art, Sungkyunkwan University, Seoul 03063, Republic of Korea; 13142146707@163.com; 3 College of Media And Communication, Jiangsu Second Normal University, Nanjing 210003, China

**Keywords:** northern goshawk optimization, off-center learning strategy, partitioned learning strategy, Bernstein-weighted learning strategy, mural image segmentation, 65K05

## Abstract

In the process of mural protection and restoration, using optimization algorithms for image segmentation is a common method for restoring mural details. However, existing optimization-based image segmentation methods often lack image segmentation quality. To alleviate the aforementioned issues, this paper proposes a mural image segmentation algorithm based on OPBNGO by integrating the Northern Goshawk Optimization (NGO) algorithm with the off-center learning strategy, partitioned learning strategy, and Bernstein-weighted learning strategy. In OPBNGO, firstly, the off-center learning strategy is proposed, which effectively improves the global search ability of the algorithm by utilizing biased center individuals. Secondly, the partitioned learning strategy is introduced, which achieves a better balance between the exploration and development phases by applying diverse learning methods to the population. Finally, the Bernstein-weighted learning strategy is proposed, which effectively improves the algorithm’s development performance. Subsequently, the OPBNGO algorithm is applied to solve the image segmentation problem for eight mural images. Experimental results show that it achieves a winning rate of over 96.87% in terms of fitness function value, achieves a winning rate of over 93.75% in terms of FSIM, SSIM, and PSNR metrics, and can be considered a promising mural image segmentation algorithm.

## 1. Introduction

As an indispensable component of world cultural heritage, murals embody profound historical and cultural connotations and values [1,2,3,4,5,6,7,8,9,10]. However, during the process of long-term preservation and maintenance, mural image information undergoes degradation, posing significant challenges to the scientific conservation of cultural heritage [11,12,13,14,15,16,17,18,19,20,21]. Currently, mural conservation scholars are dedicated to scientifically and effectively preserving and restoring murals through various means to inherit their historical, artistic, and scientific values [22,23,24,25,26,27]. In the process of mural image restoration, the identification of degraded details in images is of paramount importance [28]. Image segmentation is a common technique that effectively identifies inconspicuous details by dividing an image into multiple regions based on different regional attributes [29]. Currently, metaheuristic-based image segmentation algorithms have garnered considerable attention due to their structural simplicity, practicality, and computational efficiency [30].

Metaheuristic algorithms are computationally lightweight algorithms with efficient search capabilities, inspired by various natural phenomena and biological behaviors observed in nature. Common classification methods categorize them into four types: evolution-based algorithms, swarm-based algorithms, human-based algorithms, and physics and chemistry-based algorithms [31]. Among recent evolution-based algorithms, notable examples include the Liver Cancer Algorithm (LCA) [32], Electric Eel Foraging Optimization (EEFO) algorithm [33], Barnacles Mating Optimizer (BMO) algorithm [34], and Greylag Goose Optimization (GGO) algorithm [35]. These algorithms primarily update optimal solutions by simulating natural evolutionary behaviors of organisms, leveraging their inherent advantage of high search parallelism. This parallelism has enabled the application of LCA, EEFO, BMO, and GGO to solve diverse engineering problems, including medical feature selection, hydropower plant gate control, optimal reactive power dispatch, and UCL feature selection. Due to their low computational complexity, these algorithms have demonstrated excellent engineering applicability. Recent population-based algorithms include the Snake Optimizer (SO) [31], Genghis Khan Shark Optimizer (GKSO) [36], Sled Dog Optimizer (SDO) [37], and Zebra Optimization Algorithm (ZOA) [38]. The key advantage of these algorithms lies in their collective search mechanism, which maintains high population diversity during the search process, enabling thorough exploration of the solution space. Leveraging these strengths, SO, GKSO, SDO, and ZOA have been successfully applied to mechanical parameter design, constrained optimization problems in real-world scenarios, robotic path planning, and photovoltaic parameter optimization. These applications demonstrate their exceptional solution stability and high-precision optimization capabilities. Recent human-inspired algorithms include the Aitken optimizer (ATK) [39], Divine Religions Algorithm (DRA) [40], and Quadratic Interpolation Optimization (QIO) algorithm [41]. The primary advantage of these algorithms lies in their simulation of human-related cognitive processes, which endows them with adaptive capabilities. This adaptability accelerates the localization of optimal solutions during the search process. Currently, ATK, DRA, and QIO have been successfully applied to specific engineering tasks, including engineering design, neural network parameter tuning, and operation management of energy storage systems in microgrids. These applications demonstrate their high optimization stability. Recent physics and chemistry-inspired algorithms include the Tornado Optimizer with Coriolis Force (TOC) [42], Snow Ablation Optimizer (SAO) [43], Light Spectrum Optimizer (LSO) [44], and Chernobyl Disaster Optimizer (CDO) [45]. These algorithms draw inspiration from physical and chemical phenomena, offering advantages such as simple structures, low computational costs, and strong local exploitation capabilities. They have been successfully applied to industrial process planning, photovoltaic parameter identification, engineering design problems, and high-dimensional real-world optimization tasks. Experimental results demonstrate their superior solution speed and precision. Due to their excellent search efficiency, researchers have extensively applied these algorithms to solve image segmentation problems.

In recent years, researchers have developed numerous efficient image segmentation algorithms based on metaheuristic algorithms. For instance, Houssein et al. proposed a Snake Optimization Algorithm with Opposition-Based Learning (SO-OBL) to address deficiencies in CT scan image segmentation for liver diseases. Subsequent experiments demonstrated that SO-OBL excels in global optimization and multi-level image segmentation, achieving excellent results in FSIM, SSIM, and PSNR metrics and outperforming various metaheuristic algorithms, thereby proving its efficiency and accuracy in computer-aided diagnostic systems for images [46]. Lian et al., aiming to enhance segmentation similarity and image quality in image segmentation problems, introduced a novel Parrot Optimizer (PO). Inspired by the behavior of African Gray Parrots, PO possesses robust global and local search capabilities. Experiments on disease diagnosis and medical image segmentation problems confirmed that PO is a promising and competitive image segmentation algorithm, significantly improving image segmentation quality [47]. Qiao et al. proposed a hybrid algorithm combining the Arithmetic Optimization Algorithm and Harris Hawks Optimizer (AOA-HHO) for solving multi-level threshold image segmentation problems. Through image segmentation at seven different threshold levels, AOA-HHO demonstrated superior performance over comparison algorithms in terms of segmentation accuracy, fitness function value, peak signal-to-noise ratio, and structural similarity, making it a promising image segmentation method [48]. Addressing challenges in medical image segmentation, Yuan et al. proposed an efficient metaheuristic algorithm called the Artemisinin Optimization (AO) Algorithm. By balancing global search and local exploitation capabilities, AO exhibits strong optimization performance. Experiments on six threshold level segmentations of fifteen real breast cancer pathology images confirmed that AO outperforms eight high-performing algorithms in segmentation accuracy, feature similarity index, peak signal-to-noise ratio, and structural similarity index values, making it an efficient image segmentation method [49]. To address deficiencies in current image segmentation performance, Chen et al. proposed a novel Poplar Optimization Algorithm (POA) to alleviate shortcomings in image segmentation problems. By enhancing population diversity, the POA significantly improves optimization performance. Experiments on multi-threshold segmentations of six standard images confirmed that the POA is a metaheuristic algorithm with excellent image segmentation performance [50]. Wang et al., addressing the limitations of the Whale Optimization Algorithm (WOA) in solving image segmentation problems, such as its weak local search capability, susceptibility to local optima, and inability to balance exploration and exploitation, proposed a WOA with Crossover and Removal of Similarity (CRWOA). Using the CRWOA for multi-level threshold segmentation of 10 grayscale images, experimental results showed that the CRWOA outperformed five comparison algorithms in convergence and segmentation quality, demonstrating the superiority of the proposed algorithm [51]. Arunita et al. proposed an image segmentation method combining the Lévy–Cauchy Arithmetic Optimization Algorithm (LCAOA) and Rough K-Means (RKM). By introducing Lévy flight and Cauchy distribution to balance exploration and exploitation phases and employing opposition-based learning to enhance algorithm efficiency, experiments on multiple images demonstrated excellent segmentation performance across various image types, particularly in traditional color images, oral pathology images, and leaf images, with high feature similarity and accuracy [52]. Wang et al. proposed a multi-threshold segmentation method for breast cancer images based on an improved Dandelion Optimization Algorithm (IDOA) to address deficiencies of traditional threshold segmentation methods in complex structures and fuzzy cell boundaries. Experiments on multi-threshold segmentation of breast cancer images showed that the method, by combining opposition-based learning and an IDOA, achieves more accurate lesion area segmentation and performs well on multiple performance metrics, validating the effectiveness and superiority of the proposed method [53]. Mostafa et al. proposed an Improved Chameleon Swarm Algorithm (ICSA) to address medical image segmentation challenges. By integrating an optimal stochastic mutation strategy utilizing Lévy, Gaussian, and Cauchy distribution functions, ICSA mitigates premature convergence and local optima entrapment issues commonly encountered in medical image segmentation, thereby enhancing the quality of segmented images. However, the algorithm’s runtime efficiency was not explicitly addressed [54]. Hashim et al. introduced a modified Exponential Distribution Optimizer (mEDO) to improve multilevel image segmentation. The mEDO overcomes the limitations of the original EDO in handling complex image segmentation tasks by incorporating phasor operators and an adaptive optimal mutation strategy. Experimental results indicate that mEDO outperforms other optimizers in terms of convergence speed and accuracy, demonstrating superior performance in multi-threshold image segmentation tasks [55]. The detailed information on image segmentation algorithms is summarized in Table 1.

The aforementioned image segmentation methods based on optimization algorithms demonstrate the effectiveness of optimization algorithms in the field of image segmentation and also confirm that the introduction of improvement strategies has a positive effect on enhancing image segmentation performance. However, existing image segmentation methods based on optimization algorithms still face issues such as getting trapped in local optimal segmentation threshold combinations when addressing specific image segmentation problems, such as mural image segmentation, leading to poor image segmentation quality. Therefore, there is an urgent need to propose a novel and robust image segmentation tool with efficient image segmentation performance to alleviate this deficiency. Fortunately, the Northern Goshawk Optimization (NGO) [56] algorithm is a novel optimization algorithm that has efficient search performance. Hence, in this paper, the NGO algorithm is introduced into the field of image segmentation to achieve better mural image segmentation results. However, with the increasing dimension and complexity of optimization problems and the growing complexity of image information, the original NGO algorithm exhibits issues such as insufficient exploration capability, insufficient development capability, and an imbalance between exploration and development phases, resulting in decreased optimization accuracy when solving complex optimization problems and mural image segmentation problems. Based on the aforementioned issues, this paper proposes an improved NGO algorithm with efficient performance, called OPBNGO, by integrating the off-center learning strategy, partitioned learning strategy, and Bernstein-weighted learning strategy. In the OPBNGO algorithm, firstly, to address the issue of insufficient exploration capability of the NGO algorithm when solving mural image segmentation problems, the off-center learning strategy is introduced. By combining the fitness function values of different individuals, the off-center individuals of the population are summarized. Subsequently, the population is guided by these off-center individuals, enhancing the algorithm’s global search capability. Secondly, to tackle the imbalance between the exploration phase and the development phase of the NGO algorithm when solving mural image segmentation and high-dimensional optimization problems, the partitioned learning strategy is proposed. The population are divided into exploration-phase individuals and development-phase individuals based on their fitness function values, and diverse learning methods are applied to them, achieving better balance between the exploration and development phases and improving the algorithm’s ability to escape from local suboptimal solutions. Finally, to address the issue of decreased optimization accuracy caused by the insufficient development capability of the NGO algorithm when solving mural image segmentation problems, the Bernstein-weighted learning strategy is proposed. By leveraging the weighted properties of Bernstein polynomials, individuals with different properties are weighted to form weighted individuals, which are then used to guide the population individuals, effectively improving the algorithm’s development performance and promoting optimization accuracy. The main contributions of this paper are as follows:

- The off-center learning strategy was integrated by incorporating the fitness function values of individuals, specifically by guiding the population with off-center individuals, effectively enhancing the algorithm’s global search capability.
- The partitioned learning strategy was included by integrating fitness function values, and by applying diverse learning methods to the population, better balance between the exploration and development phases was achieved, thereby improving the algorithm’s ability to escape from local suboptimal solutions.
- The Bernstein-weighted learning strategy was applied by leveraging the weighted properties of second-order Bernstein polynomials, and by guiding the population with weighted individuals, the algorithm’s development performance was effectively improved.
- Building upon the NGO algorithm, an enhanced NGO algorithm named OPBNGO was proposed by integrating the aforementioned three learning strategies.
- The OPBNGO algorithm was employed to solve multi-threshold segmentation problems on eight mural images, achieving remarkable results in terms of fitness function values, PSNR, SSIM, and FSIM metrics, thereby confirming that the OPBNGO algorithm is a promising image segmentation method.

The subsequent work plan of this paper is as follows. Section 2 introduces the mathematical model and implementation logic of the NGO algorithm. In Section 3, the off-center learning strategy, partitioned learning strategy, and Bernstein-weighted learning strategy are proposed, and based on these, the implementation logic of the OPBNGO algorithm is presented. Section 4 applies the OPBNGO algorithm to solve the multi-threshold segmentation problem of eight mural images, verifying its potential in the field of mural image segmentation. Section 5 provides the research conclusions of this paper and outlines future work plans.

## 2. Mathematical Model of Northern Goshawk Optimization

In this section, the main focus is on the mathematical modeling of the NGO algorithm. The NGO algorithm is a novel optimization algorithm inspired by the hunting behavior of the northern goshawk, which consists of two main behaviors. In the first behavior, it locates its prey by orienting itself to its position over a wide area and moving quickly towards it. In the second behavior, after reaching an area very close to the prey, it attacks the prey for the purpose of hunting. In the resulting NGO algorithm, the exploration phase corresponding to the algorithm is formed by simulating the first behavior, and the exploitation phase corresponding to the algorithm is formed by simulating the second behavior. In the follow-up, the exploration and exploitation phases of the NGO algorithm will be mathematically modeled in detail, and the execution logic of the NGO algorithm in solving the optimization problem will be given.

### 2.1. Population Initialization

When solving an optimization problem using the NGO algorithm, an initialization population operation is first required. Each individual in the population represents a candidate solution to the optimization problem to be solved, and at the beginning of the algorithm iteration, the individuals are generated by randomly generating them within the constraints of the upper and lower bounds of the variables of the optimization problem to be solved, and the initialized population that is formed is represented as Equation (1):

(1) $$X={\left[\begin{array}{c} X_{1}\\ \vdots \\ X_{i}\\ \vdots \\ X_{N} \end{array}\right]}_{N\times Dim}={\left[\begin{array}{ccccc} x_{1,1} & \cdots & x_{1,j} & \cdots & x_{1,Dim}\\ \vdots & \ddots & \vdots & \ddots & \vdots \\ x_{i,1} & \cdots & x_{i,j} & \cdots & x_{i,Dim}\\ \vdots & \ddots & \vdots & \ddots & \vdots \\ x_{N,1} & \cdots & x_{N,j} & \cdots & x_{N,Dim} \end{array}\right]}_{N\times Dim}$$

where N denotes the population size, Dim denotes the dimension of the variable to be optimized, $X_{i}$ denotes the information of the ith individual, and $x_{i,j}$ denotes the information of the jth dimension of the ith individual, where the value of $x_{i,j}$ is randomly generated in the interval [lb,ub] at the beginning of the iteration, lb and ub denote the lower and upper bound constraints of the variable to be optimized, respectively. As mentioned earlier, an individual in the population represents a solution to the problem to be optimized; the fitness function values are used to differentiate the quality of the solutions when solving the optimization problem; and each individual corresponds to a fitness function value, denoted as Equation (2):

(2) $$F={\left[\begin{array}{c} F_{1}=F(X_{1})\\ \vdots \\ F_{i}=F(X_{i})\\ \vdots \\ F_{N}=F(X_{N}) \end{array}\right]}_{N\times 1}$$

where F(·) denotes the objective function of the problem to be optimized; and $F_{i}$ denotes the value of the fitness function corresponding to the ith individual.

### 2.2. Exploration Phase

During the population initialization phase, a set of initial candidate solutions is generated. Subsequently, the hunting behavior of the northern goshawk is emulated to optimize the solutions, making them more suitable for solving optimization problems. In the hunting behavior, the first step is to locate the prey area in a vast space, which corresponds to the exploration phase in algorithm implementation. The primary goal of the exploration phase is to identify regions that may contain potential optimal solutions. During this phase, the positions of individuals are updated using Equation (3):

(3) $$x_{i,j}^{new}=\left\{\begin{array}{ll} x_{i,j}+r\cdot \left(P_{j}-I\cdot x_{i,j}\right), & F_{P}<F_{i}\\ x_{i,j}+r\cdot \left(x_{i,j}-P_{j}\right), & F_{P}\geq F_{i} \end{array}\right.$$

where $x_{i,j}^{new}$ represents the new state of the jth dimension information of the ith individual after being updated through the exploration phase, $x_{i,j}$ represents the jth dimension information of the ith individual, r represents a random number generated within the interval [0, 1], I represents a constant randomly selected from the set {1, 2}, P represents a random individual different from individual $X_{i}$, $P_{j}$ represents the jth dimension information of the randomly selected individual P, $F_{P}$ represents the fitness function value corresponding to the randomly selected individual P, and $F_{i}$ represents the fitness function value corresponding to the ith individual. After undergoing the update of their new states during the exploration phase, individuals obtain a new individual $X_{i}^{new}$. Subsequently, it is necessary to retain the generated new state to ensure that the quality of the individuals is not compromised. Equation (4) is used to preserve individual information:

(4) $$X_{i}=\left\{\begin{array}{ll} X_{i}^{new}, & F_{i}^{new}<F_{i}\\ X_{i}, & F_{i}^{new}\geq F_{i} \end{array}\right.$$

### 2.3. Exploitation Phase

During the exploration phase of the aforementioned algorithm, the optimal solution region was identified. Subsequently, the second phase of simulating the hunting behavior of the northern goshawk, which involves attacking the prey at a very short distance, is utilized to implement the exploitation behavior of the algorithm. This allows the algorithm to further exploit potential local optimal regions, thereby ensuring the precision of the algorithm when solving optimization problems. Assuming that the northern goshawk attacks the prey when the distance to the prey is R, Equation (5) is used to represent the update method of individual positions during the exploitation phase of the algorithm:

(5) $$x_{i,j}^{new}=x_{i,j}+R\cdot \left(2\cdot r-1\right)\cdot x_{i,j}$$

where $x_{i,j}^{new}$ represents the new state of the jth dimension information of the ith individual after being updated during the exploitation phase. r denotes a random number generated within the interval [0, 1]. R signifies the distance from the northern goshawk to its prey, as represented by Equation (6):

(6) $$R=0.02\cdot (1-\frac{t}{T})$$

where t represents the current number of algorithm iterations, and T represents the maximum number of algorithm iterations. After updating the new state of individuals during the exploitation phase, a new individual $X_{i}^{new}$ is obtained. Subsequently, it is necessary to retain the generated new state to ensure that the quality of the individuals is enhanced. Equation (4) is used to preserve individual information.

### 2.4. Implementation of the NGO Algorithm

**Figure 1:**
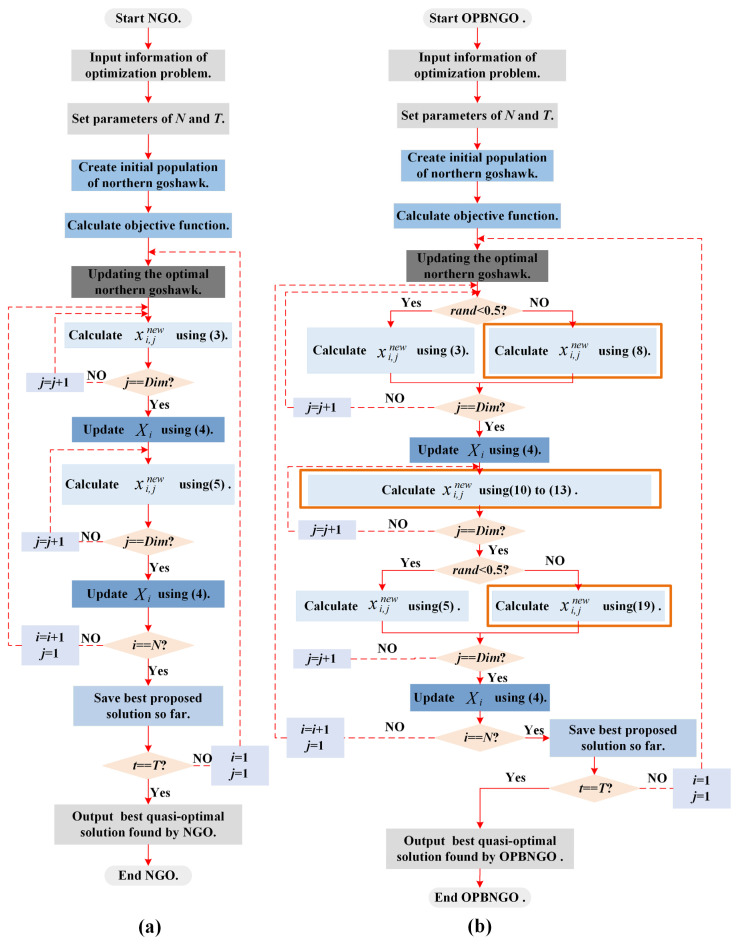
Execution flowchart of algorithm. (**a**). NGO algorithm. (**b**). OPBNGO algorithm.

In the previous section, detailed mathematical modeling was conducted for the initialization phase, exploration phase, and exploitation phase involved in the NGO algorithm. Consequently, in this section, the execution logic of the NGO algorithm when addressing practical optimization problems is summarized, integrating the aforementioned theoretical knowledge. The execution pseudocode is represented as Algorithm 1, and the execution flowchart is depicted in Figure 1a.
**Algorithm 1:** Pseudo code for NGO algorithm**Input:** Population size (N), Dimension of the optimization problem (Dim), Upper bound of the optimization problem (Ub) and lower bound (Lb), Maximum number of iterations (T).
**Output:** Global best solution ($X_{best}$).
1. Initialize the population using Equation (1) and calculate the individual fitness function values of the population.2. ***for*** t=1:T3.    ***for*** i=1:N4.       ***exploration phase***5.       ***for*** j=1:Dim6.            Calculate the jth dimensional new state of the ith individual using Equation (3).7.       ***end for***8.       Use Equation (4) to preserve the new state of individual $X_{i}$.9.       ***Exploitation Phase***10.     ***for*** j=1:Dim11.          Calculate the jth dimensional new state of the ith individual using Equation (5).12.     ***end for***13.     Use Equation (4) to preserve the new state of individual $X_{i}$.14.  ***end for***15.  Save the global best solution $X_{best}$.16.***end for***17.Output the global best solution $X_{best}$ obtained by solving the optimization problem using the NGO algorithm.

## 3. Mathematical Model of Improved Northern Goshawk Optimization

The previous section mainly reviewed the mathematical model and execution logic of the NGO algorithm. However, when solving high-dimensional complex optimization problems and image segmentation issues, the NGO algorithm, due to the limitations of its strategy, suffers from insufficient exploration capability, insufficient exploitation capability, and an imbalance between the exploration and exploitation phases. This leads to a lack of population diversity and a tendency to fall into local suboptimal solutions when solving optimization problems, resulting in poor optimization accuracy and local stagnation. To effectively enhance the performance of the NGO algorithm in solving high-dimensional complex optimization problems and image segmentation issues, this section addresses its existing deficiencies and proposes corresponding improvement strategies to enhance the algorithm’s optimization performance. First, to address the insufficient exploration capability when solving image segmentation issues, this section introduces the off-center learning strategy. By considering the fitness function values of different individuals, the off-center individuals of the population are identified. Guiding the population through these off-center individuals enhances population diversity during the execution process and strengthens the algorithm’s global search capability. Second, to address the imbalance between the exploration and exploitation phases when solving image segmentation and high-dimensional optimization problems, this section proposes the partitioned learning strategy. By segmenting the population into exploration and exploitation individuals based on fitness function values and updating them through different learning methods, better balance between exploration and exploitation phases is achieved, enhancing the algorithm’s ability to escape local suboptimal solutions. Finally, to address the insufficient exploitation capability leading to decreased optimization accuracy when solving image segmentation issues, this section introduces the Bernstein-weighted learning strategy. Using the weighted properties of Bernstein polynomials, individuals of different natures are weighted to form weighted individuals, which are then used to guide the population, effectively improving the algorithm’s exploitation performance and promoting optimization accuracy. The following section will detail the mathematical models of the off-center learning strategy, partitioned learning strategy, and Bernstein-weighted learning strategy proposed in this section, and propose an improved NGO algorithm (OPBNGO) based on the above strategies.

### 3.1. Off-Center Learning Strategy

The original NGO algorithm faces a deficiency in global exploration capability when solving image segmentation and high-dimensional complex optimization problems, leading to a decrease in population diversity during the iterative process. This is detrimental to the further expansion of algorithm performance and application extension. To alleviate this issue, there is an urgent need to propose a novel and efficient global search strategy. Deng et al. [57] pointed out that guiding the population using the average point of individuals within the population can effectively enhance the algorithm’s global exploration performance, providing significant inspiration for the global exploration strategy proposed in this section. In this section, to further enhance the algorithm’s global exploration capability, an off-center learning strategy is proposed based on the aforementioned idea of average point guidance. Traditional average point guidance does not consider the nature of different individuals; thus, although the algorithm has strong global search capabilities, the local exploitation capability during the iterative process is not guaranteed, resulting in certain deficiencies in optimization accuracy. Based on this consideration, in this section, different fitness function values of individuals are utilized, with the main idea being that the mean point of individuals in the population should be closer to high-quality individuals. This ensures that when guiding the population through the off-center point, both the global search capability of the population and the local exploitation capability of the algorithm are maintained. Combining the aforementioned theoretical ideas forms the off-center weighted point, represented by Equation (7):

(7) $$X_{off}=\sum_{i=1}^{N}\frac{F_{max}-F_{i}+\varepsilon }{\sum_{j=1}^{N}(F_{max}-F_{i}+\varepsilon )}\cdot X_{i}$$

where $X_{off}$ represents the off-center weighted point, $F_{max}$ denotes the maximum fitness function value corresponding to the individuals in the population, and ε signifies a very small positive number, the purpose of which is to ensure that the denominator is not zero; in this section, $\varepsilon =10^{-15}$. The traditional average point does not consider individual attributes, which can easily lead to insufficient convergence behavior of the algorithm in the later stages of iteration. In contrast, the off-center weighted point pointed out in this section tends to explore areas with higher quality individuals, while also incorporating the concept of the average point. This effectively enhances the algorithm’s global search capability and overcomes the deficiency of insufficient convergence behavior in the later stages of iteration. Additionally, Equation (7) also shows that individuals with higher quality, corresponding to smaller fitness function values, have greater weighting applied, favoring exploration of high-quality areas.

**Figure 2:**
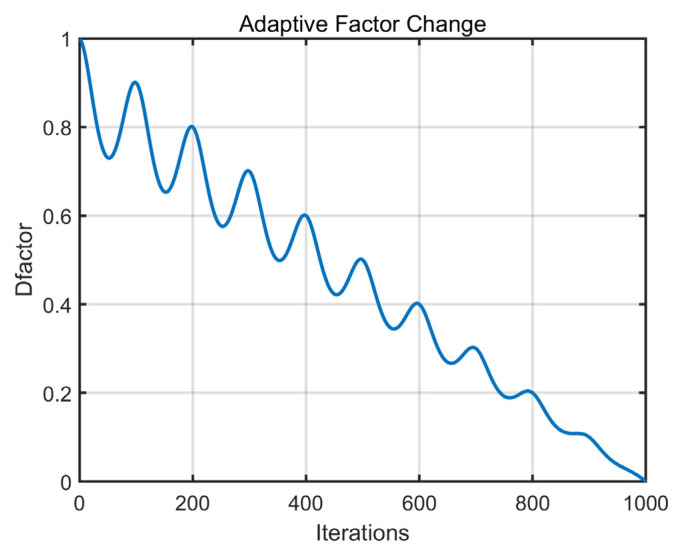
The change chart of the adaptive factor.

The aforementioned theoretical analysis highlighted the advantages of the off-center weighted point. Subsequently, this off-center weighted point is used to guide individual positions to enhance the algorithm’s actual global exploration capability. During the guidance process, considering that global exploration behavior should gradually decrease as the iteration progresses to better improve the algorithm’s convergence speed and accuracy, an adaptive factor Dfactor, is incorporated into the guidance mechanism. The formed off-center learning strategy is represented by Equation (8):

(8) $$x_{i,j}^{new}=x_{i,j}+\cos((1-(\frac{t}{T}))\cdot \pi )\cdot Dfactor\cdot (x_{off,j}-x_{i,j})$$

where $x_{off,j}$ denotes the jth dimensional information of the off-center weighted point. The adaptive factor Dfactor is represented by Equation (9), and the image visualization is shown in Figure 2:

(9) $$Dfactor=\frac{1-\frac{t}{T}}{1+A\cdot \sin{\left(\omega \cdot \frac{t}{T}\right)}^{2}}$$

where the values of A and ω are 0.3 and 10π, respectively.

From the figure, it can be observed that as iterations progress, Dfactor decreases from 1 to 0 in an adaptively decaying manner. This property ensures that the off-center learning strategy effectively enhances the algorithm’s global search capability during the early iterations. As iterations continue, to ensure the convergence accuracy of the algorithm, this learning process gradually diminishes. In summary, the off-center learning strategy proposed in this section can effectively strengthen the algorithm’s global search ability, thereby improving the algorithm’s performance in solving image segmentation problems. Unlike conventional global exploration strategies, the off-center learning strategy proposed in this section is guided not by a global average point but by an off-center point. Moreover, instead of relying on ordinary linear factor control, this strategy introduces a novel sinusoidal nonlinear factor for guidance. This innovation enables adaptive modulation of the guidance process, thereby enhancing the algorithm’s global search capabilities.

### 3.2. Partitioned Learning Strategy

**Figure 3:**
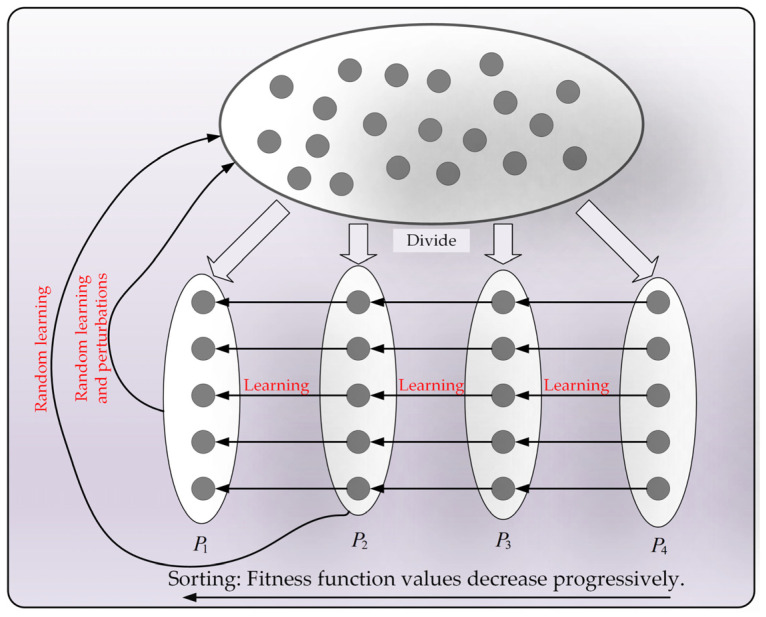
The schematic diagram of the partitioned learning strategy.

The original NGO algorithm exhibits an imbalance between the exploration and exploitation phases when addressing image segmentation and high-dimensional optimization problems. This imbalance hinders the algorithm’s ability to effectively escape from local suboptimal traps, leading to deficiencies in the selection of threshold combinations and the precision of optimization problems. Wu et al. [58] indicated that categorizing individuals in the population based on fitness function values into multiple groups can achieve a certain balance between the exploration and exploitation phases of the algorithm. Inspired by this, a novel partitioned learning strategy is proposed in this section, aimed at reasonably balancing the exploration and exploitation phases of the algorithm to enhance its ability to escape local optimal traps. In the partitioned learning strategy, first, the entire population is divided into four individual sets, $P_{1}$, $P_{2}$, $P_{3}$, and $P_{4}$, based on fitness function values. Subsequently, individuals in $P_{4}$ learn from those in $P_{3}$, and individuals in $P_{3}$ learn from those in $P_{2}$, primarily enhancing the algorithm’s exploitation capability. Individuals in $P_{2}$ learn from those in $P_{1}$ and random individuals in the population, while individuals in $P_{1}$ undergo small-scale Gaussian–Cauchy perturbations and learn from random individuals, mainly ensuring the algorithm’s global exploration capability. By applying different learning methods to individuals of different natures, a good balance between the global search and local exploitation phases of the algorithm is achieved. To visually represent this learning process, the execution of the partitioned learning strategy is visualized in Figure 3. Subsequently, the four learning methods are introduced in detail below.

Firstly, individuals from segment $P_{4}$ learn from those in segment $P_{3}$, a process represented by Equation (10):

(10) $$x_{i,j}^{new}=x_{i,j}+rand\cdot (1-\frac{t}{T})\cdot (x_{P3,j}-x_{i,j})$$

where rand denotes a random number within the interval [0, 1], and $x_{P3,j}$ represents the jth dimensional value of an individual from segment $P_{3}$. Secondly, individuals from segment $P_{3}$ learn from those in segment $P_{2}$, a process represented by Equation (11):

(11) $$x_{i,j}^{new}=x_{i,j}+rand\cdot e^{l}\cdot \cos(2\pi l)\cdot (x_{P2,j}-x_{i,j})$$

where l represents a value randomly selected from the set {−1, 1}, and $x_{P2,j}$ denotes the jth dimensional value of an individual from segment $P_{2}$. Thirdly, individuals from segment $P_{2}$ learn from those in segment $P_{1}$, as well as from random individuals within the population, a process represented by Equation (12):

(12) $$\begin{array}{c} x_{i,j}^{new}=x_{i,j}+0.5\cdot \left(1-\frac{t}{T}\right)\cdot \left(1+0.3\cdot sin\left(10\pi \cdot t\right)\right)\\ \cdot (x_{P1,j}-x_{i,j})+0.5\cdot rand\cdot (x_{rand,j}-x_{i,j}) \end{array}$$

where $x_{P1,j}$ denotes the jth dimensional value of an individual from segment $P_{1}$, and $x_{rand,j}$ represents the jth dimensional value of a random individual different from $X_{i}$ within the population. Finally, individuals from segment $P_{1}$ learn from random individuals in the population and undergo Gaussian–Cauchy perturbations, a process represented by Equation (13):

(13) $$x_{i,j}^{new}\left\{\begin{array}{l} x_{i,j}+\left(1+0.3\cdot arctan(10\pi \cdot t)\right)\cdot (x_{rand,j}-x_{i,j})\;\;if\;rand<0.5\\ x_{i,j}\cdot (1+\tau_{1}\cdot \mathrm{cauchy}(0,\sigma^{2})+\tau_{2}\cdot \mathrm{gaussian}(0,\sigma^{2}))\;\;otherwise \end{array}\right.$$

where $\mathrm{cauchy}(0,\sigma^{2})$ represents a random number that follows the standard Cauchy distribution, $\mathrm{gaussian}(0,\sigma^{2})$ represents a random number that follows the standard Gaussian (normal) distribution, $\tau_{1}$ takes the value of $1-{\left(t/T\right)}^{2}$, and $\tau_{2}$ takes the value of ${\left(t/T\right)}^{2}$. X is represented by Equation (14):

(14) $$\sigma =\frac{F_{min}-F_{r}}{F_{r}}$$

where $F_{min}$ denotes the minimum fitness function value corresponding to an individual in the population, while $F_{r}$ represents the fitness function value of a randomly selected individual within the population. By segmenting individuals in the population based on fitness function values and applying diverse learning methods, the algorithm achieves a good balance between the global exploration phase and the local exploitation phase, enhancing its ability to escape from local optimal traps. Unlike conventional balance-oriented strategies, the partitioned learning strategy proposed in this section divides the population into multiple regions based on fitness function values. Subsequently, it applies adaptive learning to individuals with distinct characteristics. This approach offers greater rationality, thereby enhancing the algorithm’s balance and improving its ability to escape local optima traps.

### 3.3. Bernstein-Weighted Learning Strategy

**Figure 4:**
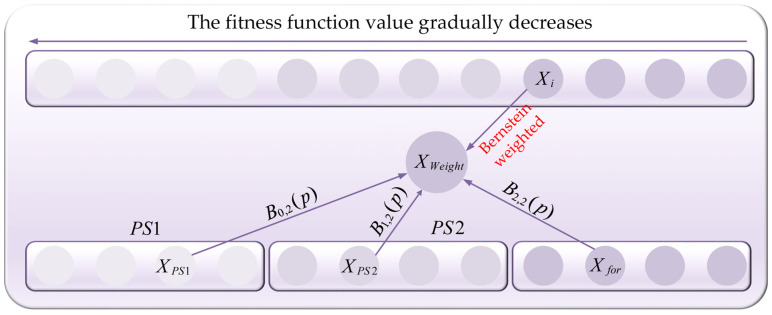
The schematic diagram of the Bernstein-weighted learning strategy.

The original NGO algorithm exhibits a deficiency in local exploitation when tackling high-dimensional optimization and image segmentation challenges. This shortcoming prevents the algorithm from further refining local optima once they are identified, resulting in a loss of optimization precision. Consequently, there is an urgent need in this section to introduce a high-quality local exploitation strategy to enhance the NGO algorithm’s capabilities and improve its segmentation and optimization accuracy. Zhang et al. [59] have noted that individuals can effectively strengthen the algorithm’s local exploitation by learning from those with superior performance. Inspired by this, to further boost the NGO algorithm’s local exploitation and achieve higher optimization precision, a strategy based on the weighting of candidates using second-order Bernstein polynomials is proposed in this section. This strategy weights individuals from a pool of better candidates and random individuals and then uses these weighted individuals to guide the search of the current individual. The pool of better candidates is defined as the set of individuals with fitness function values lower than that of the current individual $X_{i}$. To provide an intuitive understanding of this concept, the strategy based on second-order Bernstein polynomial weighting is visualized as shown in Figure 4, and the formation process of this strategy will be detailed subsequently.

**Figure 5:**
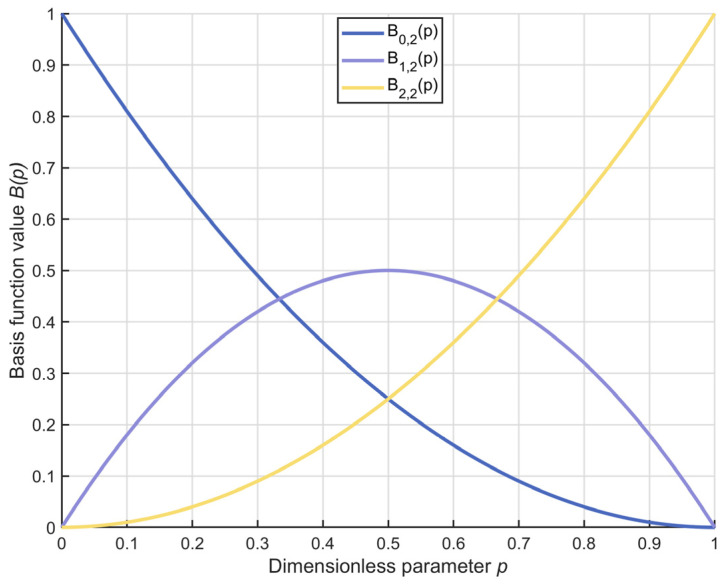
The change chart of second-order Bernstein polynomials.

Firstly, the nth-order Bernstein polynomial is defined by Equation (15):

(15) $$B_{w,n}(p)=C_{n}^{w}\cdot p^{w}\cdot {\left(1-p\right)}^{n-p}$$

where p represents the probability of success in an experiment, with 0≤p≤1; n denotes the total number of precise trials; and w signifies the number of successful trials within those precise trials, where w=1,2,…,n. C represents the binomial coefficient, calculated using Equation (16):

(16) $$C_{n}^{w}=\frac{n!}{w!(n-w)!}$$

where the “!” denotes factorial operation. For the second-order Bernstein polynomial, n takes the value of 2, and w can be 0, 1, or 2. According to Equations (15) and (16), when w=0 and n=2, it can be derived that $B_{0,2}(p)={\left(1-p\right)}^{2}$; when w=1 and n=2, it can be derived that $B_{1,2}(p)=2\cdot p\cdot (1-p)$; and when w=2 and n=2, it can be derived that $B_{2,2}(p)=p^{2}$. In summary, the second-order Bernstein polynomial consists of three polynomials, as shown in Equation (17):

(17) $$\left\{\begin{array}{l} B_{0,2}(p)={\left(1-p\right)}^{2}\\ B_{1,2}(p)=2\cdot p\cdot (1-p)\\ B_{2,2}(p)=p^{2} \end{array}\right.$$

where, to better analyze the properties of the second-order Bernstein polynomials, they are visualized as shown in Figure 5. The horizontal axis represents p ranging from 0 to 1, and the vertical axis represents the values of the Bernstein polynomial functions as p changes. From the curves, it can be observed that when 0≤p≤1, the values of $B_{0,2}(p)$, $B_{1,2}(p)$, and $B_{2,2}(p)$ are also confined between 0 and 1. Moreover, for any value of p, the sum of the function values of these three polynomials always equals 1. This property is then utilized to generate Bernstein-weighted individuals, defining $p={\left(t/T\right)}^{2}$, where p nonlinearly increases from 0 to 1 with the increase in the number of function evaluations.

The process of generating Bernstein-weighted individuals is as follows: Firstly, individuals in the pool of superior candidate solutions are evenly divided into two segments based on their fitness function values, ordered from smallest to largest. These segments are defined as segments PS1 and PS2. Subsequently, one random individual is drawn from each of these segments, denoted as $X_{PS1}$ and $X_{PS2}$. Then, another random individual is selected from the population, excluding the pool of superior candidate solutions, defined as $X_{for}$. The Bernstein polynomials $B_{0,2}(p)$, $B_{1,2}(p)$, and $B_{2,2}(p)$ are assigned as weighting coefficients to individuals $X_{PS1}$, $X_{PS2}$, and $X_{for}$, respectively, to generate the weighted individual $X_{Weight}$, represented by Equation (18):

(18) $$X_{Weight}=B_{0,2}(p)\cdot X_{PS1}+B_{1,2}(p)\cdot X_{PS2}+B_{2,2}(p)\cdot X_{for}$$

Subsequently, the generated Bernstein-weighted individual $X_{Weight}$ is used to guide the individuals, as represented in Equation (19):

(19) $$x_{i,j}^{new}=x_{i,j}+\left(1-\frac{t}{T}\right)\cdot \left(1+0.3\cdot arctan{\left(10\pi \cdot t\right)}^{2}\right)\cdot (x_{Weight,j}-x_{i,j})$$

By employing the Bernstein-weighted learning strategy to guide individuals, the local exploitation capability of the algorithm is significantly enhanced. Additionally, the introduction of a certain degree of randomness in this strategy reduces the probability of the algorithm falling into local optimal traps, further optimizing convergence precision. Unlike conventional strategies that rely solely on the optimal individual to guide the population and enhance local exploitation capabilities, the Bernstein-weighted learning strategy proposed in this section leverages the parameter-weighted nature of Bernstein polynomial factors to generate representative weighted individuals for guiding the population. The advantage of this approach lies in its ability to utilize the broader representational capacity of weighted individuals when the optimal individual is trapped in a local optimum. By doing so, the weighted individuals can guide the algorithm to escape local optima, thereby simultaneously improving local exploitation efficiency and preserving global search capabilities. This dual enhancement ultimately leads to higher convergence accuracy.

### 3.4. Implementation of the OPBNGO Algorithm

The initial NGO algorithm encounters several issues when solving high-dimensional optimization and image segmentation problems, such as a deficiency in global exploration, an imbalance between the global exploration and local exploitation phases, and insufficient local exploitation. These issues result in a loss of population diversity, a propensity to fall into local optima, and a decrease in optimization accuracy. To mitigate these challenges, this paper introduces an off-center learning strategy to enhance the algorithm’s global search ability, leveraging off-center individuals based on their fitness values. Additionally, a partitioned learning strategy is proposed to balance the exploration and exploitation phases by segmenting the population and applying distinct learning methods. Lastly, a Bernstein-weighted learning strategy is presented to improve the algorithm’s exploitation performance and optimize accuracy by weighting individuals based on Bernstein polynomials. These strategies are integrated into an enhanced NGO algorithm, termed OPBNGO, with Algorithm 2 providing its pseudocode and Figure 1b depicting its execution flowchart.
**Algorithm 2:** Pseudo code for OPBNGO algorithm**Input:** Population size (N), Dimension of the optimization problem (Dim), Upper bound of the optimization problem (Ub) and lower bound (Lb), Maximum number of iterations (T).
**Output:** Global best solution ($X_{best}$).
1. Initialize the population using Equation (1) and calculate the individual fitness function values of the population.2. ***for*** t=1:T3.    ***for*** i=1:N4.       ***exploration phase***5.       ***for*** j=1:Dim6.            ***if*** rand<0.57.                    Calculate the jth dimensional new state of the ith individual using Equation (3).8.            ***else***9.                    Calculate the jth dimensional new state of the ith individual using Equation (8).10.          ***end if***11.     ***end for***12.     Use Equation (4) to preserve the new state of individual $X_{i}$.13.     ***Balance Phase***14.     ***for*** j=1:Dim15.          Calculate the jth dimensional new state of the ith individual using Equations (10)–(13).16.     ***end for***17.     Use Equation (4) to preserve the new state of individual $X_{i}$.18.     ***Exploitation Phase***19.     ***for*** j=1:Dim20.          ***if*** rand<0.521.                  Calculate the jth dimensional new state of the ith individual using Equation (5).22.          ***else***23.                  Calculate the jth dimensional new state of the ith individual using Equation (19).24.          ***end if***25.     ***end for***26.     Use Equation (4) to preserve the new state of individual $X_{i}$.27.   ***end for***28.   Save the global best solution $X_{best}$.29.***end for***30.Output the global best solution $X_{best}$ obtained by solving the optimization problem using OPBNGO algorithm.

### 3.5. Time Complexity of the OPBNGO Algorithm

In this section, the time complexity of the OPBNGO algorithm is mainly analyzed. The OPBNGO algorithm mainly includes the population initialization stage and the algorithm iteration stage. Among them, the basic operation of the algorithm is to calculate the fitness function value of individuals. Therefore, the computational complexity of the algorithm initialization stage is O(N). In the algorithm iteration stage, a total of T iterations are required, including the exploration stage, partitioned learning stage, and exploitation stage. Therefore, the fitness function value of individuals in the iteration stage is evaluated 3N times, and the computational complexity of the algorithm iteration stage is O(3N·T). Therefore, the computational complexity of the OPBNGO algorithm is O(N·(1+3T)).

## 4. Results and Discussion on Mural Image Segmentation

In this section, we primarily assess the performance of the proposed OPBNGO algorithm in solving multi-threshold segmentation problems for mural images. Thresholding mural images primarily involves separating different regions within the mural image, such as the mural itself, the background, and damaged areas. This is beneficial for promoting the conservation and restoration of murals. Through multi-threshold segmentation, areas with different attributes are separated to better capture image information, allowing researchers to more effectively restore and protect the images. In this section, we mainly use the Otsu method as the objective function to identify the optimal image segmentation thresholds. The core idea is to maximize the inter-class variance between different regions of the image to confirm the optimal threshold combination. Therefore, the objective function here refers to the core concept of the Otsu method, which is to maximize the inter-class variance. By searching with the OPBNGO algorithm, we obtain the threshold combination that maximizes the inter-class variance between different image regions, effectively segmenting the different areas of the mural image. In the following sections, we will detail the Otsu method and evaluate the performance of the OPBNGO algorithm based on the Otsu method in solving multi-threshold segmentation problems for mural images.

### 4.1. Concept of Otsu Thresholding Technique

In this section, the concept of the Otsu method is primarily introduced. The core idea behind the Otsu method for image segmentation is to maximize the inter-class variance between different regions of the image, effectively separating distinct areas. This section will detail the concept further. Assuming the pixel matrix of the image to be segmented is represented as I, and there are L grayscale levels within the image, with $n_{i}$ being the number of pixels at grayscale level i, the total number of pixels N in image I can be calculated using Equation (20):

(20) $$N=\sum_{i=0}^{L-1}n_{i}$$

Subsequently, the probability distribution $P_{i}$ for the grayscale level i is derived and calculated using Equation (21):

(21) $$P_{i}=\frac{n_{i}}{N},\;\;\;i=0,1,\ldots ,L-1$$

where $P_{i}\geq 0,\;P_{0}+P_{1}+\cdots +P_{L-1}=1$. The number of thresholds used for image segmentation is set to k. Assuming the threshold for image segmentation is t, the threshold t divides the image into two regions: the region with pixel grayscale values in the interval [1, t] is classified as the target region, and the region with pixel grayscale values in the interval [t + 1, *L*−1] is classified as the background region. The ratio of the number of pixels in the classified target region to the total number of pixels in the image is defined as $\omega_{0}$, and the average grayscale value of the region is $\mu_{0}$. The ratio of the number of pixels in the classified background region to the total number of pixels in the image is defined as $\omega_{1}$, and the average grayscale value of the region is $\mu_{1}$. The average grayscale value of the entire image is μ, and the variance between different image segmentation regions is ν. Based on the above assumptions, $\omega_{0}$, $\mu_{0}$, $\omega_{1}$, $\mu_{1}$, μ, and ν are calculated using Equations (22)–(27), respectively:

(22) $$\omega_{0}=\sum_{i=0}^{t}P_{i}$$

(23) $$\mu_{0}=\sum_{i=0}^{t}\frac{iP_{i}}{\omega_{0}}$$

(24) $$\omega_{1}=\sum_{i=t+1}^{L-1}P_{i}$$

(25) $$\mu_{1}=\sum_{i=t+1}^{L-1}\frac{iP_{i}}{\omega_{1}}$$

(26) $$\mu =\sum_{i=0}^{k-1}\omega_{i}\mu_{i}\;=\sum_{i=0}^{L-1}iP_{i}$$

(27) $$v(t)=\omega_{0}{\left(\mu_{0}-\mu \right)}^{2}+\omega_{1}{\left(\mu_{1}-\mu \right)}^{2}=\omega_{0}\omega_{1}{\left(\mu_{0}-\mu_{1}\right)}^{2}$$

Subsequently, the optimal image segmentation threshold $t_{best}$ is calculated using Equation (28):

(28) $$t_{\mathrm{best}}={\arg\max}_{0\leq t\leq L}\left[v(t)\right]$$

Subsequently, the inter-class variance for multiple threshold values k is calculated using Equation (29):

(29) $$\begin{array}{ll} v(t_{1},t_{2},\ldots ,t_{k}) & =\omega_{0}\omega_{1}{\left(\mu_{0}-\mu_{1}\right)}^{2}+\omega_{0}\omega_{2}{\left(\mu_{0}-\mu_{2}\right)}^{2}+\cdots \\ & +\omega_{0}\omega_{k}{\left(\mu_{0}-\mu_{k}\right)}^{2}+\omega_{1}\omega_{2}{\left(\mu_{1}-\mu_{2}\right)}^{2}+\cdots \\ & +\omega_{1}\omega_{3}{\left(\mu_{1}-\mu_{3}\right)}^{2}+\cdots +\omega_{k-1}\omega_{k}{\left(\mu_{k-1}-\mu_{k}\right)}^{2} \end{array}$$

where $\omega_{i}$ and $\mu_{i}$ are calculated using Equations (30) and (1), respectively.

(30) $$\omega_{i-1}=\sum_{i=t_{i-1}+1}^{t_{i}}P_{i},\;1\leq i\leq k+1$$

(31) $$\mu_{i-1}=\sum_{i=t_{i-1}+1}^{t_{i}}\frac{iP_{i}}{\omega_{i-1}},\;1\leq i\leq k+1$$

Assuming the best segmentation threshold combination for the image is $T_{\mathrm{best}}=(t_{1}^{*},t_{2}^{*},\ldots ,t_{k}^{*})$, the optimal threshold combination is calculated using Equation (32):

(32) $$T_{\mathrm{best}}=\arg\max_{0\leq t_{1}\leq t_{2}\leq \cdots \leq \cdots \leq t_{k}}\left[v(t_{1},t_{2},\ldots ,t_{k})\right]$$

### 4.2. Experimental Results Analysis of Mural Image Segmentation

**Figure 6:**
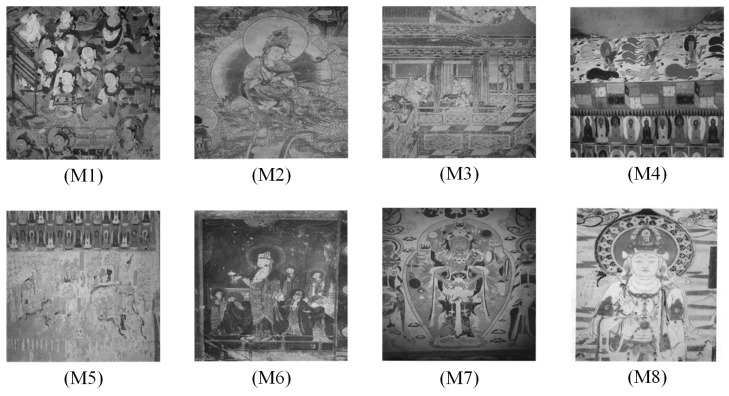
The information of eight mural images.

In this section, the performance of the OPBNGO algorithm based on Otsu in solving the problem of multi-threshold image segmentation of murals is mainly evaluated. Through image segmentation experiments on eight mural images, the image information is shown in Figure 6, and the performance of the proposed algorithm is comprehensively evaluated. Meanwhile, to ensure fairness, the maximum number of function iterations is set to 100, the population size is set to 40, and each experiment is independently executed 30 times without repetition. In addition, to better evaluate the performance of the OPBNGO algorithm based on Otsu in mural image segmentation, it is compared with six highly efficient algorithms. The specific parameter settings of the comparison algorithms are shown in Table 2. Subsequently, through statistical analysis of the commonly used evaluation indicators in the field of image segmentation, namely the peak signal-to-noise ratio (PSNR) [60], the structural similarity index measure (SSIM) [61], and the feature similarity index measure (FSIM) [62], the analysis mainly includes the mean and standard deviation of the statistical results, so as to comprehensively evaluate the performance of the OPBNGO algorithm based on Otsu in multi-threshold segmentation of mural images. Among them, the higher the PSNR and SSIM values are, the lower the distortion of the segmented image will be, and the higher the FSIM value is, the lower the error rate of image segmentation will be. The above evaluation indicators will be analyzed in detail below. Furthermore, to offer an intuitive visualization of the final image segmentation outcomes and streamline the subsequent analysis of segmentation performance metrics, Table 3 displays the segmentation results achieved by the OPBNGO when the number of segmentation thresholds is set to 2, 4, 6, and 8, respectively.

#### 4.2.1. Strategy Effectiveness Analysis of Mural Image Segmentation

**Figure 7:**
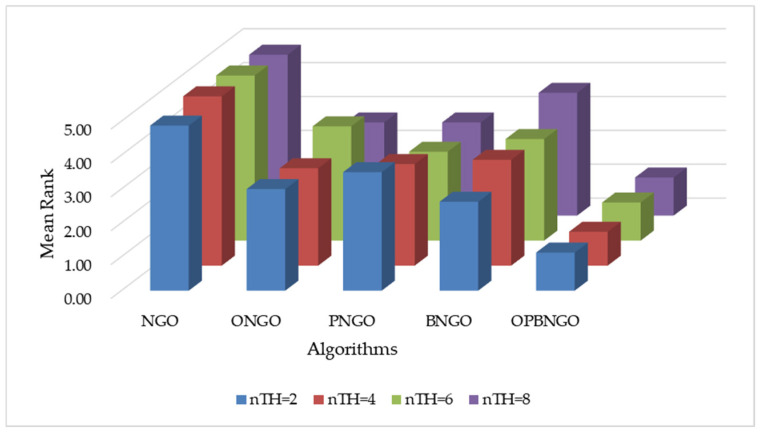
Figure illustrating results of strategy ablation experiments.

This section primarily focuses on validating the effectiveness of the three learning strategies incorporated into the OPBNGO algorithm to demonstrate that each strategy can effectively enhance the algorithm’s performance. First, we define the ONGO algorithm as the NGO algorithm augmented with the off-center learning strategy, the PNGO algorithm as the NGO algorithm integrated with the partitioned learning strategy, and the BNGO algorithm as the NGO algorithm enhanced with the Bernstein-weighted learning strategy. Based on these definitions, we employ NGO, ONGO, PNGO, BNGO, and OPBNGO algorithms to solve the mural image segmentation problems for eight images with threshold counts of 2, 4, 6, and 8, respectively, thereby analyzing the effectiveness of the strategies. The experimental results are illustrated in Figure 7, where “Mean Rank” denotes the average ranking based on the mean fitness values across the eight mural image segmentation tasks. As depicted in the figure, under varying threshold conditions, ONGO, PNGO, and BNGO all exhibit lower bar heights compared to NGO, indicating that the incorporation of a single learning strategy effectively enhances the performance of mural image segmentation, thereby validating the effectiveness of the strategies. Furthermore, it is noteworthy that OPBNGO, which integrates all three learning strategies simultaneously, demonstrates even lower bar heights than ONGO, PNGO, and BNGO. This suggests that OPBNGO achieves superior performance in mural image segmentation, confirming that the simultaneous integration of the three learning strategies into NGO further elevates the algorithm’s segmentation capabilities. In summary, these findings demonstrate that introducing each learning strategy individually into the NGO algorithm effectively enhances its performance, while integrating all three strategies concurrently maximizes the algorithm’s performance gains.

#### 4.2.2. Fitness Value Metric Analysis of Mural Image Segmentation

**Figure 8:**
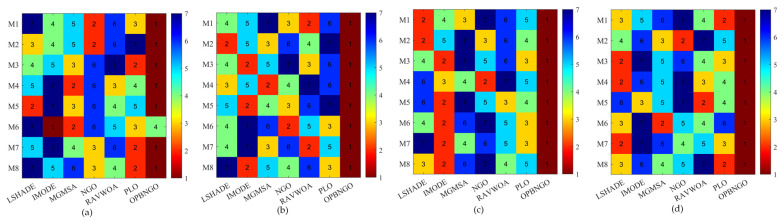
Ranking plot of fitness function values for mural image segmentation. (**a**). nTH = 2. (**b**). nTH = 4. (**c**). nTH = 6. (**d**). nTH = 8.

**Figure 9:**
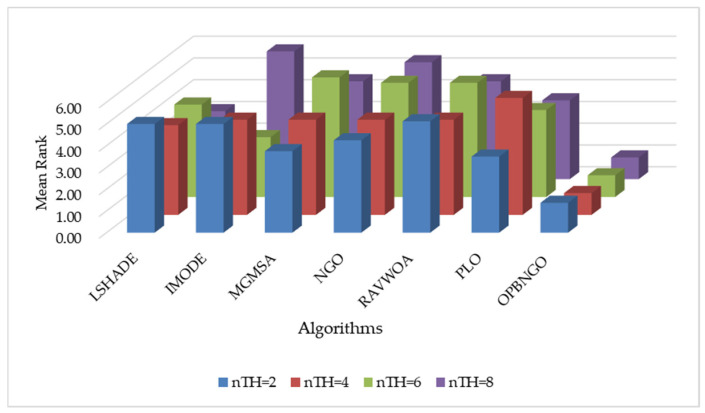
Bar chart of average rankings of fitness function values for mural image segmentation.

This section primarily focuses on analyzing the fitness function values of the OPBNGO algorithm for mural image segmentation. The detailed experimental results are presented in Supplementary Table S1, encompassing the mean values and standard deviations derived from 30 independent trials. To provide a more intuitive demonstration of the algorithm’s superiority, Figure 8 illustrates the ranking of the algorithm’s fitness function values across the segmented mural images, while Figure 9 depicts the algorithm’s average ranking of fitness function values for varying numbers of segmentation thresholds. Specifically, in Figure 8, subplots (a), (b), (c), and (d) correspond to the rankings of the algorithm’s fitness function values when the number of segmentation thresholds is set to two, four, six, and eight, respectively.

As illustrated in Figure 8, when the number of segmentation thresholds is set to two, the OPBNGO algorithm achieves the optimal fitness function values for seven out of eight mural image segmentation tasks, yielding a success rate of 87.5%. For segmentation thresholds of four, six, and eight, the OPBNGO algorithm attains the optimal fitness function values across all eight mural image segmentation tasks, achieving a 100% success rate. These experimental results confirm that the OPBNGO algorithm benefits from the advanced strategies integrated into its design, enabling efficient exploration of the threshold combination space and ensuring a high degree of exploratory capability in the solution space. Furthermore, as depicted in Figure 9, when the number of segmentation thresholds is two, four, six, or eight, OPBNGO exhibits lower bar heights (indicating superior performance) and achieves a lower average ranking of fitness function values compared to the benchmark algorithms. In summary, the integration of these strategies into the OPBNGO algorithm facilitates effective exploration of segmentation threshold combinations, enhancing its performance in mural image segmentation. The algorithm demonstrates superior segmentation outcomes compared to the benchmarks, positioning it as a promising approach for mural image segmentation.

#### 4.2.3. PSNR Metric Analysis of Mural Image Segmentation

**Figure 10:**
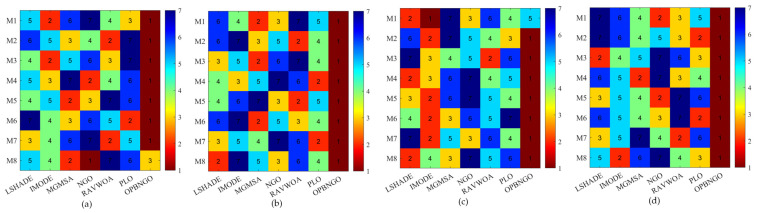
Ranking plot of PSNR values for mural image segmentation. (**a**). nTH = 2. (**b**). nTH = 4. (**c**). nTH = 6. (**d**). nTH = 8.

**Figure 11:**
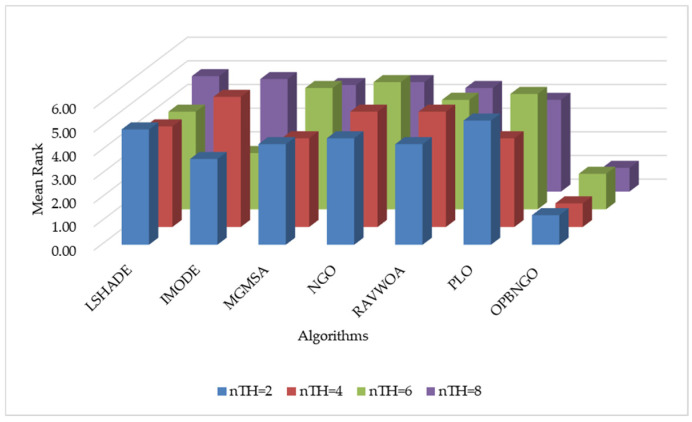
Bar chart of average rankings of PSNR values for mural image segmentation.

In this section, we primarily analyze the peak signal-to-noise ratio (PSNR) achieved by the OPBNGO algorithm for mural image segmentation to evaluate the distortion level post segmentation. The detailed experimental results for the PSNR are presented in Supplementary Table S2, encompassing the mean values and standard deviations derived from 30 independent trials. To facilitate the analysis of the PSNR metric, Figure 10 illustrates the ranking of the algorithm’s PSNR values when addressing the segmentation of eight mural images, while Figure 11 depicts the algorithm’s average ranking of PSNRs across varying numbers of segmentation thresholds. Specifically, in Figure 10, subplots (a), (b), (c), and (d) correspond to the rankings of the algorithm’s PSNR values when the number of segmentation thresholds is set to two, four, six, and eight, respectively.

As depicted in Figure 10, when the number of segmentation thresholds is set to two, the OPBNGO algorithm achieves the optimal PSNR values for seven out of eight mural image segmentation tasks, yielding a success rate of 87.5%. For a segmentation threshold count of four, the OPBNGO algorithm attains the optimal PSNR values across all eight mural image segmentation tasks, achieving a 100% success rate. When the number of segmentation thresholds is six, OPBNGO again achieves the optimal PSNR values for seven out of eight tasks, with an 87.5% success rate. Finally, for a segmentation threshold count of eight OPBNGO secures the optimal PSNR values for all eight mural image segmentation tasks, reaching a 100% success rate. Concurrently, as shown in Figure 11, across experiments with varying numbers of segmentation thresholds, OPBNGO exhibits lower bar heights (indicating superior performance) and achieves a lower average ranking of PSNR values compared to the benchmark algorithms. These experimental results confirm that, when addressing mural image segmentation tasks, the OPBNGO algorithm demonstrates a lower image distortion rate compared to the benchmarks. It effectively captures image details while maximizing the quality of the segmented images. Furthermore, these findings validate that the OPBNGO algorithm is more precise in balancing noise suppression and detail preservation when identifying optimal segmentation parameters, thereby enhancing the overall mural segmentation performance.

#### 4.2.4. SSIM Metric Analysis of Mural Image Segmentation

**Figure 12:**
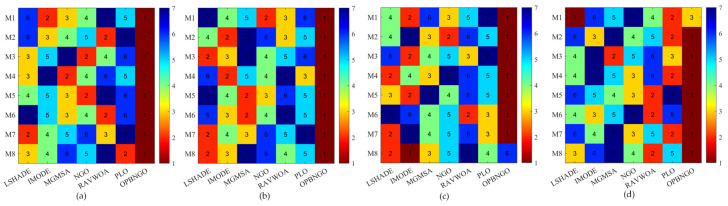
Ranking plot of SSIM values for mural image segmentation. (**a**). nTH = 2. (**b**). nTH = 4. (**c**). nTH = 6. (**d**). nTH = 8.

**Figure 13:**
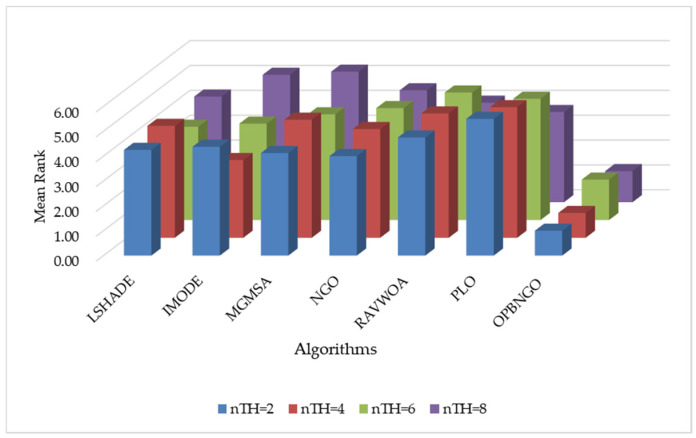
Bar chart of average rankings of SSIM values for mural image segmentation.

In this section, we primarily analyze the structural similarity index measure (SSIM) achieved by the OPBNGO algorithm for mural image segmentation to evaluate the structural similarity between the segmented images and their original counterparts. The detailed experimental results for the SSIM are presented in Supplementary Table S3, encompassing the mean values and standard deviations derived from 30 independent trials. To facilitate the analysis of the SSIM metric, Figure 12 illustrates the ranking of the algorithm’s SSIM values when addressing the segmentation of eight mural images, while Figure 13 depicts the algorithm’s average ranking of SSIM across varying numbers of segmentation thresholds. Specifically, in Figure 12, subplots (a), (b), (c), and (d) correspond to the rankings of the algorithm’s SSIM values when the number of segmentation thresholds is set two, four, six, and eight, respectively.

As illustrated in Figure 12, when the number of segmentation thresholds is set to two and 4, the OPBNGO algorithm achieves the optimal SSIM values across all eight mural image segmentation tasks, attaining a 100% success rate compared to the benchmark algorithms. For segmentation threshold counts of six and eight, OPBNGO secures the optimal SSIM values for seven out of eight tasks, yielding an 87.5% success rate. Concurrently, as depicted in Figure 13, across experiments with varying numbers of segmentation thresholds, OPBNGO exhibits lower bar heights (indicating superior performance) and achieves a lower average ranking of SSIM values compared to the benchmark algorithms. These experimental results demonstrate that the OPBNGO algorithm effectively searches for optimal combinations of segmentation thresholds when addressing mural image segmentation tasks, achieving a favorable trade-off among evaluation metrics. It outperforms the benchmark algorithms by obtaining the highest SSIM values, which implies that the segmented images exhibit high structural similarity to the original images in terms of key visual features such as structural information, contrast, and luminance. This indicates that OPBNGO effectively preserves the boundaries, textures, and details of objects within the images, thereby validating it as a promising approach for mural image segmentation.

#### 4.2.5. FSIM Metric Analysis of Mural Image Segmentation

**Figure 14:**
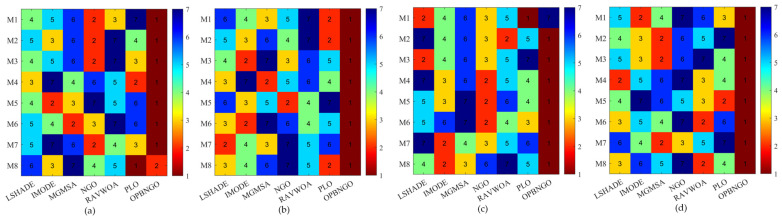
Ranking plot of FSIM values for mural image segmentation. (**a**). nTH = 2. (**b**). nTH = 4. (**c**). nTH = 6. (**d**). nTH = 8.

**Figure 15:**
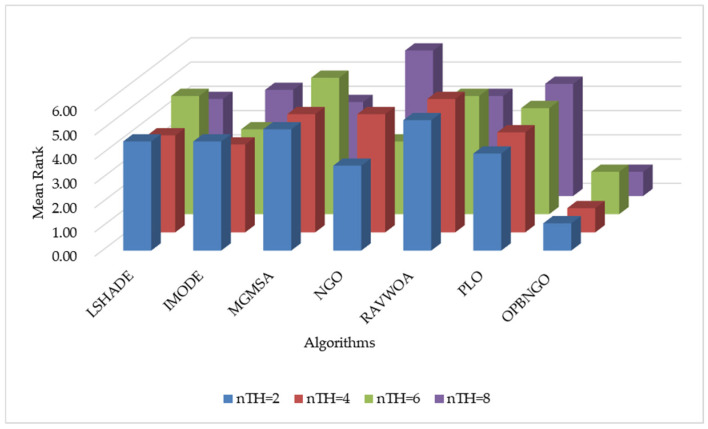
Bar chart of average rankings of FSIM values for mural image segmentation.

In this section, we primarily analyze the feature similarity index measure (FSIM) achieved by the OPBNGO algorithm for mural image segmentation to evaluate the feature similarity between the segmented images and their original counterparts. The detailed experimental results for the FSIM are presented in Supplementary Table S4, encompassing the mean values and standard deviations derived from 30 independent trials. To facilitate the analysis of the FSIM metric, Figure 14 illustrates the ranking of the algorithm’s FSIM values when addressing the segmentation of eight mural images, while Figure 15 depicts the algorithm’s average FSIM ranking across varying numbers of segmentation thresholds. Specifically, in Figure 14, subplots (a), (b), (c), and (d) correspond to the rankings of the algorithm’s FSIM values when the number of segmentation thresholds is set to two, four, six, and eight, respectively.

As depicted in Figure 14, when the number of segmentation thresholds is set to two and six, the OPBNGO algorithm achieves the optimal FSIM values for seven out of eight mural image segmentation tasks, yielding an 87.5% success rate compared to the benchmark algorithms. For segmentation threshold counts of four and eight, OPBNGO attains the optimal FSIM values across all eight tasks, achieving a 100% success rate. Concurrently, as illustrated in Figure 15, across experiments with varying numbers of segmentation thresholds, OPBNGO exhibits lower bar heights (indicating superior performance) and achieves a lower average ranking of FSIM values compared to the benchmark algorithms. These experimental results demonstrate that the OPBNGO algorithm is capable of achieving high-quality segmentation of mural images, effectively extracting intricate details while maximizing the preservation of the original image’s feature information. It strikes a favorable balance between detail extraction and retention. Compared to the benchmark algorithms, OPBNGO achieves the highest FSIM values, signifying greater feature similarity and lower distortion between the segmented images and their original counterparts. This indicates superior retention of critical structural elements within the images, along with enhanced preservation of edge clarity and texture definition, thereby minimizing information loss post segmentation. Collectively, these findings validate OPBNGO as an effective image segmentation methodology.

In summary, through the analysis of the fitness function values PSNR, SSIM, and FSIM in multi-threshold image segmentation, it is confirmed that the OPBNGO algorithm proposed in this paper, due to the advanced nature of its search strategy, achieves outstanding results in these four metrics. It can effectively segment mural images with the minimum image distortion rate and the highest structural similarity, making it a promising method for multi-threshold image segmentation.

#### 4.2.6. Convergence Analysis of Mural Image Segmentation

**Figure 16:**
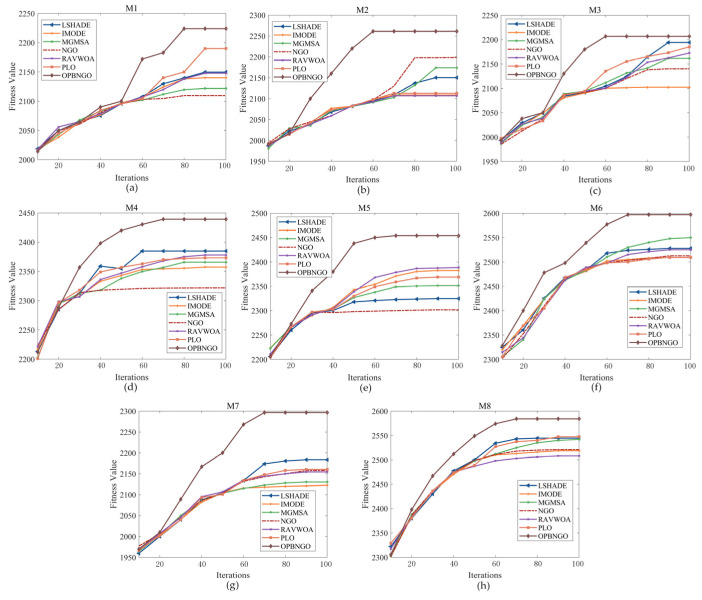
Convergence plot for mural image segmentation algorithms. (**a**). M1. (**b**). M2. (**c**). M3. (**d**). M4. (**e**). M5. (**f**). M6. (**g**). M7. (**h**). M8.

The preceding analysis of the key performance metrics for the OPBNGO algorithm in addressing mural image segmentation tasks has validated its potential as a promising approach for mural image segmentation. However, beyond segmentation accuracy, the convergence characteristics of an algorithm during image segmentation are equally critical. A robust algorithm should exhibit a faster convergence rate to ensure its practical applicability. Consequently, this section focuses on analyzing the convergence behavior of the OPBNGO algorithm when solving mural image segmentation problems. The experimental results for a segmentation threshold count of eight are illustrated in Figure 16, where the X-axis denotes the number of iterations, and the Y-axis represents the fitness function value.

As illustrated in the figure, each algorithm effectively enhances the fitness function value and, in most cases, attains a stable convergence state, thereby validating the efficacy of each algorithm in solving mural image segmentation problems. Notably, the OPBNGO algorithm establishes a notable lead after 30 iterations in the majority of scenarios. This is primarily attributed to the integration of three learning strategies, which significantly bolster the algorithm’s exploitation capabilities, enabling it to secure an early advantage during the initial iterations. As the iterations progress, the OPBNGO algorithm’s lead widens further, largely due to its superior global search capabilities, which allow it to effectively escape local optima traps in segmentation threshold combinations during the later stages of iteration. Additionally, it is evident that the OPBNGO algorithm achieves a stable convergence state after approximately 60 iterations, demonstrating a faster convergence rate compared to the benchmark algorithms. These experimental observations substantiate that the proposed OPBNGO algorithm, owing to its advanced strategies, not only achieves higher convergence accuracy but also exhibits a faster convergence speed, thereby qualifying it as a practical and effective approach for mural image segmentation.

#### 4.2.7. Computational Time Analysis of Mural Image Segmentation

**Figure 17:**
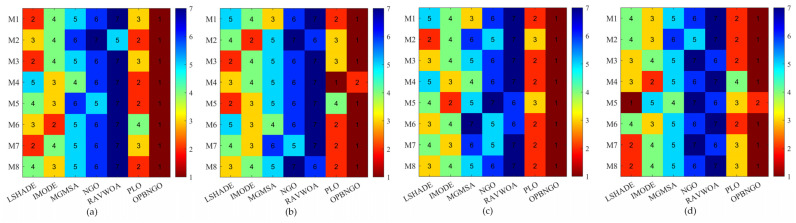
Ranking plot of runtime for mural image segmentation. (**a**). nTH = 2. (**b**). nTH = 4. (**c**). nTH = 6. (**d**). nTH = 8.

**Figure 18:**
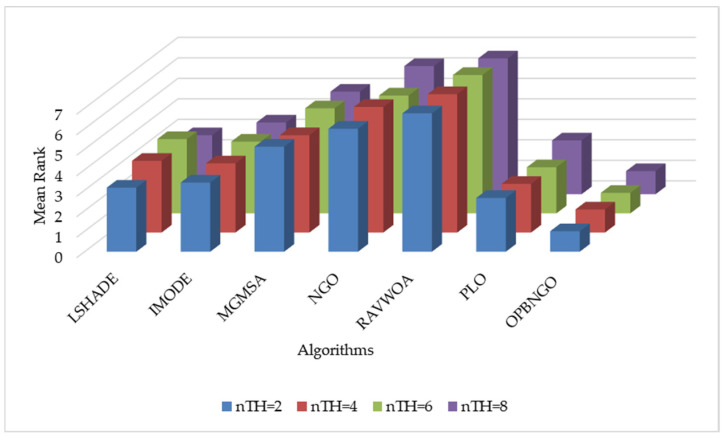
Bar chart of average rankings of runtime for mural image segmentation.

In this section, we primarily focus on quantifying the actual runtime performance of the algorithm when addressing mural image segmentation tasks. The detailed numerical statistics are presented in Supplementary Table S5. To facilitate discussion, Figure 17 illustrates the ranking of algorithms’ runtime metrics when solving the segmentation problem for eight mural images, while Figure 18 depicts the average runtime rankings of the algorithms across varying numbers of segmentation thresholds. Specifically, in Figure 17, subplots (a), (b), (c), and (d) correspond to the runtime rankings of the algorithm when the number of segmentation thresholds is set to two, four, six, and eight, respectively.

As illustrated in Figure 17, the OPBNGO algorithm achieves the top runtime ranking across all eight mural image segmentation tasks when the number of segmentation thresholds is set to two and six, attaining a 100% success rate. For threshold counts of four and eight, OPBNGO ranks first in seven out of eight tasks, yielding an 87.5% success rate. Furthermore, Figure 18 demonstrates that, across varying numbers of segmentation thresholds, the OPBNGO algorithm consistently exhibits lower bar heights, indicating shorter actual runtimes. These experimental analyses substantiate that the proposed OPBNGO algorithm achieves faster execution speeds when solving mural image segmentation problems, effectively reducing computational overhead and minimizing resource wastage. Consequently, it qualifies as a promising methodology for mural image segmentation.

## 5. Conclusions and Future Works

In this paper, to address the issue of decreased optimization accuracy caused by insufficient global exploration capability, inadequate exploitation capability, and imbalance between the exploration and exploitation phases in the original NGO algorithm when solving mural image segmentation problems, an enhanced NGO algorithm called OPBNGO is proposed by integrating three learning strategies, which significantly improves the algorithm’s optimization performance. In the proposed OPBNGO algorithm, firstly, to tackle the problem of inadequate exploration capability of the NGO algorithm in solving mural image segmentation problems, an off-center learning strategy is introduced, which enhances population diversity during the algorithm’s execution and strengthens the algorithm’s global search capability. Secondly, to address the imbalance between the exploration and exploitation phases of the NGO algorithm in solving mural image segmentation, a partitioned learning strategy is proposed, which updates individual information through different learning methods, achieving better balance between the exploration and exploitation phases and improving the algorithm’s ability to escape local suboptimal solutions. Finally, to address the issue of decreased optimization accuracy caused by the inadequate exploitation capability of the NGO algorithm in solving mural image segmentation problems, a Bernstein-weighted learning strategy is proposed, which utilizes the weighted properties of Bernstein polynomials to guide population individuals using weighted individuals, effectively enhancing the algorithm’s exploitation performance and promoting optimization accuracy. Subsequently, the OPBNGO algorithm was applied to address the segmentation problem for eight mural images. The experimental results demonstrate the following:

(1) Compared to high-performing benchmark algorithms, the OPBNGO algorithm achieves a 96.87% win rate in terms of the fitness function value. (2) It attains a 93.75% win rate across the peak signal-to-noise ratio (PSNR), structural similarity index measure (SSIM), and feature similarity index measure (FSIM) metrics. These outcomes highlight the algorithm’s robust performance in mural image segmentation, as it maximizes inter-class variance in the images while preserving structural, edge, and luminance information to the greatest extent possible. Consequently, the OPBNGO algorithm yields superior image segmentation quality and reduces distortion rates, qualifying it as a promising methodology for mural image segmentation.

While the OPBNGO algorithm proposed in this study has shown promising segmentation performance on mural images, there is still room for improvement, particularly on specific images where performance limitations exist. Therefore, future work will focus on the following aspects: (1) developing tailored improvement strategies, such as leveraging reinforcement learning for dynamic strategy selection, to enhance segmentation efficacy on these challenging cases; (2) expanding the evaluation of OPBNGO beyond mural images to broader image segmentation domains for comprehensive performance testing and validation; and (3) developing a multi-objective variant to address segmentation tasks across diverse application fields, since the current OPBNGO is designed for single-objective segmentation, and thereby advancing its versatility and applicability.

## Figures and Tables

**Table 1:** Summary of current research on image segmentation algorithms.

Authors	Year	Algorithms	Main Strategies	Results
Houssein et al. [46]	2024	SO-OBL	Opposition-based learning	High search performance
Lian et al. [47]	2024	PO	Innovative development strategies	High image segmentation performance
Qiao et al. [48]	2024	AOA-HHO	Hybrid algorithm	Low image distortion rate
Yuan et al. [49]	2024	AO	Innovative exploration strategies	High structural similarity
Chen et al. [50]	2022	POA	Innovative exploration strategies	High feature similarity
Wang et al. [51]	2023	CRWOA	Crossover and similarity removal strategies	Excellent fitness function value
Arunita et al. [52]	2023	LCAOA	Lévy–Cauchy variation	High feature similarity
Wang et al. [53]	2024	IDOA	Opposition-based learning	High image segmentation performance
Mostafa et al. [54]	2024	ICSA	Lévy, Gaussian, and Cauchy perturbation strategies	High feature similarity
Hashim et al. [55]	2024	mEDO	Phasor operators and an adaptive optimal mutation strategy	Low image distortion rate

**Table 2:** Compare algorithm parameter settings on mural image segmentation.

Name	Time	Parameter Settings
LSHADE [63]	2014	$Control\;parameters:\;NP_{init}=18\cdot D,\;\;\;NP_{min}=4,\;\;\;|A|=2.6\cdot NP,\;\;\;p=0.11,\;\;\;H=6$
IMODE [64]	2020	Control parameters: D=2, arch_rate=2.6
MGSMA [65]	2022	$Variable:\;WEP=WEP_{min}+t\cdot \left(\frac{WEP_{max}-WEP_{min}}{T}\right)$
NGO [56]	2021	$Variable:\;R=0.02\cdot (1-\frac{t}{T})$
RAVWOA [66]	2022	$Variable:\;a=2\cdot (1-\frac{t}{T})$
PLO [67]	2024	$Variable:\;W_{1}=\frac{2}{\left(1+e^{-2{\left(t/T\right)}^{4}}\right)}-1,\;W_{2}=e^{-{\left(2t/T\right)}^{3}}$

**Table 3:** Visualization of mural image segmentation results.

Image	nTH = 2	nTH = 4	nTH = 6	nTH = 8
M1	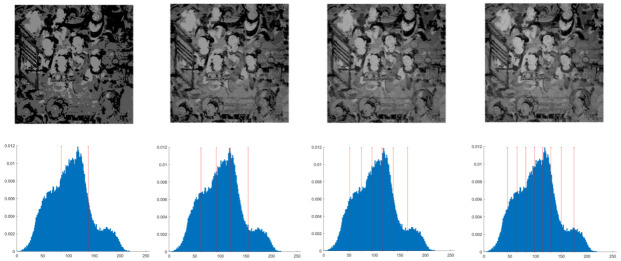			
M2	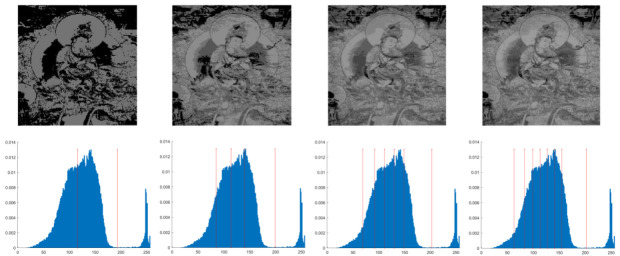			
M3	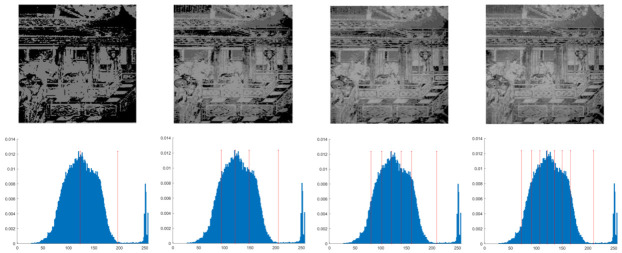			
M4	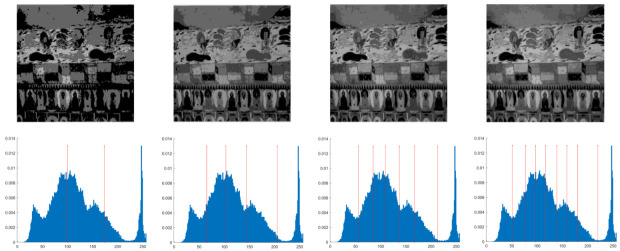			
M5	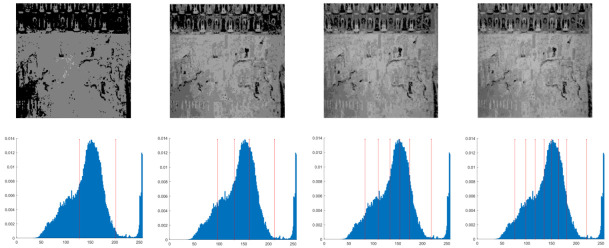			
M6	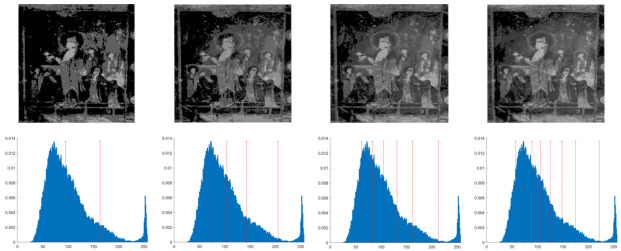			
M7	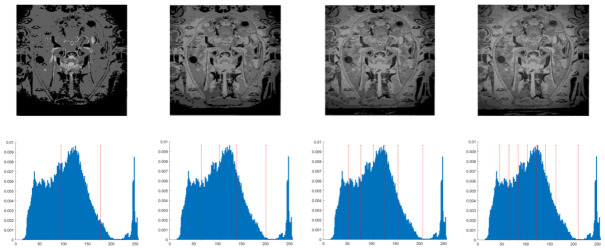			
M8	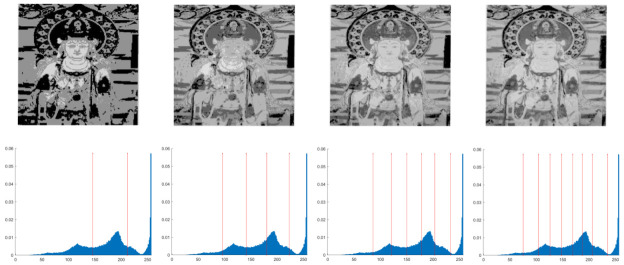			

## Data Availability

If there are legitimate and reasonable needs, one can request the relevant data from the corresponding author.

## Supplementary Materials

The following supporting information can be downloaded at: https://www.mdpi.com/article/10.3390/biomimetics10060373/s1, Table S1: The fitness function value of algorithms in mural image segmentation; Table S2: The PSNR value of algorithms in mural image segmentation; Table S3: The SSIM value of algorithms in mural image segmentation; Table S4: The FSIM value of algorithms in mural image segmentation; Table S5: The runtime of algorithms in mural image segmentation.
