# Supplementary material for: An Improved Northern Goshawk Optimization Algorithm for Mural Image Segmentation
Source: Biomimetics (Basel). 2025 Jun 5;10(6):373. doi: 10.3390/biomimetics10060373 (PMC12190925; doi:10.3390/biomimetics10060373)
Supplement: Supplementary file 1 [file biomimetics-10-00373-s001.zip › biomimetics-3626345-supplementary.pdf]

biomimetics

MDPI

Article

Supplementary Table Material (Manuscript Title: An Improved Northern Goshawk Optimization Algorithm for Mural Image Segmentation)

Supplementary Table S1. The fitness function value of algorithms in mural image segmentation.

Image	nTH	LSHADE		IMODE		MGMSA		NGO		RAVWOA		PLO		OPBNGO	
		Mean	Std	Mean	Std	Mean	Std	Mean	Std	Mean	Std	Mean	Std	Mean	Std
M1	2	1.704E+03	4.900E-01	1.770E+03	3.027E-01	1.748E+03	6.828E-02	1.806E+03	5.033E-01	1.744E+03	1.706E-02	1.792E+03	2.523E-01	1.901E+03	4.671E-03
	4	1.939E+03	4.178E-01	1.935E+03	9.489E-01	1.912E+03	2.213E-01	1.948E+03	9.590E-03	1.995E+03	7.904E-01	1.920E+03	6.343E-01	2.052E+03	4.489E-03
	6	2.069E+03	6.356E-01	2.051E+03	9.851E-01	2.054E+03	2.293E-01	2.004E+03	2.169E-01	2.028E+03	5.230E-01	2.051E+03	7.840E-01	2.109E+03	7.388E-03
	8	2.150E+03	7.587E-01	2.140E+03	8.859E-01	2.122E+03	1.894E-01	2.110E+03	6.602E-01	2.148E+03	7.270E-01	2.190E+03	9.005E-01	2.224E+03	9.170E-03
M2	2	1.783E+03	2.245E-01	1.778E+03	6.133E-02	1.774E+03	3.524E-01	1.807E+03	4.768E-01	1.773E+03	2.356E-01	1.749E+03	7.961E-01	1.934E+03	8.142E-03
	4	1.981E+03	6.014E-01	1.955E+03	4.771E-01	1.962E+03	2.627E-01	1.944E+03	2.104E-01	1.960E+03	7.808E-01	1.919E+03	7.574E-01	2.081E+03	2.251E-03
	6	2.078E+03	1.988E-01	2.036E+03	7.253E-01	2.014E+03	9.029E-02	2.058E+03	6.747E-01	2.023E+03	8.116E-01	2.036E+03	3.934E-01	2.150E+03	6.077E-03
	8	2.150E+03	6.253E-01	2.109E+03	8.601E-01	2.174E+03	2.887E-01	2.198E+03	4.410E-01	2.107E+03	7.967E-01	2.113E+03	1.183E-01	2.261E+03	2.372E-03
M3	2	1.768E+03	2.118E-01	1.731E+03	1.630E-01	1.795E+03	2.669E-01	1.710E+03	4.864E-01	1.702E+03	8.447E-01	1.896E+03	1.119E-01	1.921E+03	4.960E-03
	4	1.987E+03	8.937E-01	1.997E+03	5.062E-01	1.979E+03	9.100E-01	1.912E+03	1.101E-01	1.987E+03	5.566E-01	1.959E+03	9.261E-01	2.050E+03	4.983E-01
	6	2.030E+03	9.180E-01	2.071E+03	5.945E-01	2.004E+03	8.229E-01	2.009E+03	8.450E-01	2.006E+03	3.046E-01	2.056E+03	2.900E-01	2.145E+03	2.038E-01
	8	2.194E+03	5.343E-01	2.102E+03	2.473E-01	2.162E+03	1.282E-01	2.140E+03	4.686E-01	2.173E+03	9.660E-01	2.185E+03	8.199E-01	2.207E+03	5.850E-01
M4	2	1.955E+03	3.592E-01	1.902E+03	1.125E-01	2.091E+03	5.621E-01	1.936E+03	5.588E-01	2.038E+03	1.471E-01	1.996E+03	8.974E-01	2.190E+03	9.703E-03
	4	2.168E+03	2.359E-01	2.140E+03	8.139E-01	2.187E+03	6.409E-01	2.151E+03	4.755E-01	2.129E+03	1.468E-01	2.137E+03	4.393E-01	2.279E+03	3.467E-03
	6	2.215E+03	8.354E-01	2.275E+03	8.482E-01	2.248E+03	5.080E-01	2.291E+03	9.861E-01	2.215E+03	7.313E-01	2.219E+03	6.940E-01	2.311E+03	4.142E-04
	8	2.385E+03	1.875E-01	2.357E+03	4.459E-01	2.366E+03	4.281E-01	2.322E+03	2.171E-01	2.378E+03	2.166E-01	2.373E+03	1.741E-01	2.439E+03	6.260E-03
M5	2	2.099E+03	5.582E-01	1.941E+03	3.836E-01	2.038E+03	5.219E-01	1.948E+03	4.550E-01	2.021E+03	6.973E-01	1.988E+03	2.583E-01	2.114E+03	8.563E-03
	4	2.170E+03	2.711E-01	2.186E+03	4.293E-01	2.174E+03	5.272E-01	2.178E+03	3.436E-01	2.127E+03	5.021E-02	2.110E+03	3.436E-01	2.267E+03	4.880E-04
	6	2.227E+03	6.922E-01	2.299E+03	1.287E-01	2.201E+03	9.107E-01	2.243E+03	8.914E-01	2.279E+03	6.561E-01	2.257E+03	2.285E-01	2.330E+03	7.665E-03
	8	2.325E+03	3.355E-02	2.382E+03	3.365E-01	2.351E+03	6.478E-01	2.302E+03	8.499E-01	2.389E+03	1.915E-01	2.369E+03	2.874E-01	2.454E+03	2.753E-03
M6	2	2.119E+03	2.032E-02	2.297E+03	1.050E-01	2.274E+03	2.245E-01	2.122E+03	2.120E-01	2.161E+03	9.662E-01	2.175E+03	1.249E-01	2.174E+03	4.779E-03
	4	2.319E+03	5.489E-01	2.306E+03	4.998E-01	2.307E+03	4.472E-01	2.361E+03	4.739E-01	2.312E+03	9.146E-01	2.340E+03	1.858E-01	2.415E+03	5.170E-03
	6	2.492E+03	7.941E-01	2.495E+03	8.681E-01	2.439E+03	5.164E-01	2.418E+03	5.945E-01	2.459E+03	2.653E-01	2.495E+03	9.940E-01	2.579E+03	6.399E-03
	8	2.528E+03	6.996E-01	2.509E+03	9.580E-01	2.550E+03	4.695E-01	2.512E+03	8.643E-01	2.525E+03	6.465E-01	2.509E+03	2.131E-01	2.597E+03	4.544E-03
M7	2	1.722E+03	2.513E-01	1.702E+03	9.791E-01	1.803E+03	9.278E-01	1.833E+03	5.591E-01	1.710E+03	6.721E-01	1.852E+03	8.831E-01	1.905E+03	6.022E-03
	4	1.939E+03	6.568E-01	1.902E+03	7.030E-01	1.945E+03	2.363E-01	1.933E+03	6.664E-01	1.958E+03	7.360E-01	1.934E+03	2.883E-01	2.027E+03	4.342E-03
	6	2.005E+03	7.770E-01	2.094E+03	9.374E-01	2.066E+03	7.712E-01	2.060E+03	3.434E-01	2.065E+03	5.328E-01	2.069E+03	9.958E-01	2.163E+03	3.754E-04
	8	2.184E+03	4.106E-01	2.123E+03	8.820E-01	2.130E+03	1.220E-01	2.158E+03	9.086E-01	2.154E+03	1.445E-01	2.161E+03	5.687E-01	2.297E+03	3.848E-01
M8	2	2.105E+03	2.954E-01	2.207E+03	5.563E-01	2.173E+03	3.020E-02	2.254E+03	6.031E-01	2.243E+03	8.628E-01	2.294E+03	7.059E-01	2.302E+03	4.537E-01
	4	2.309E+03	3.037E-01	2.391E+03	6.754E-01	2.343E+03	8.585E-01	2.358E+03	7.320E-01	2.331E+03	3.645E-01	2.380E+03	3.527E-01	2.483E+03	1.818E-03
	6	2.462E+03	8.675E-01	2.463E+03	4.881E-01	2.417E+03	8.936E-01	2.401E+03	3.142E-02	2.444E+03	2.550E-01	2.424E+03	7.201E-02	2.539E+03	2.373E-03
	8	2.545E+03	9.689E-01	2.519E+03	5.733E-01	2.542E+03	1.432E-01	2.521E+03	7.327E-01	2.508E+03	3.747E-02	2.547E+03	3.943E-01	2.584E+03	5.350E-03
Friedman Rank		4.13		4.50		4.53		4.81		4.81		4.13		1.09	
Final Rank		2		4		5		6		6		2		1	

Supplementary Table S2. The PSNR value of algorithms in mural image segmentation.

Image	nTH	LSHADE		IMODE		MGMSA		NGO		RAVWOA		PLO		OPBNGO	
		Mean	Std	Mean	Std	Mean	Std	Mean	Std	Mean	Std	Mean	Std	Mean	Std
M1	2	18.475	4.505E-03	18.626	4.464E-03	18.370	4.235E-03	18.062	3.978E-03	18.547	2.382E-03	18.623	9.782E-03	19.647	3.615E-03
	4	23.100	7.853E-03	23.178	5.645E-03	23.595	2.024E-03	23.226	5.411E-03	23.090	6.694E-03	23.166	6.460E-03	24.381	4.254E-03
	6	25.903	7.631E-03	25.922	6.604E-03	25.275	9.566E-03	25.853	5.974E-03	25.491	7.891E-03	25.754	4.474E-03	25.567	9.329E-03
	8	26.116	7.018E-03	26.158	8.967E-03	26.568	8.610E-03	26.997	7.834E-03	26.678	1.880E-03	26.182	2.268E-03	27.746	2.707E-03
M2	2	18.172	7.942E-03	18.554	7.440E-03	18.705	8.259E-03	18.556	6.946E-03	18.964	6.253E-03	18.163	7.503E-03	19.806	3.400E-03

	4	23.137	5.672E-03	23.093	3.029E-03	23.664	5.392E-03	23.230	1.014E-03	23.916	2.291E-03	23.496	2.254E-03	24.366	9.031E-03
	6	25.319	8.699E-03	25.958	9.027E-03	25.081	1.306E-03	25.332	4.667E-03	25.337	3.012E-03	25.744	4.126E-03	26.843	4.002E-03
	8	27.022	5.208E-03	27.460	1.785E-03	27.556	7.018E-03	27.514	8.844E-03	27.809	5.966E-03	27.999	2.250E-03	28.282	7.337E-03
M3	2	18.698	6.651E-03	18.767	3.358E-03	18.693	8.784E-03	18.546	3.228E-03	18.731	7.457E-03	18.229	1.292E-03	19.196	9.471E-03
	4	23.786	3.473E-03	23.532	2.049E-03	23.931	1.567E-03	23.512	1.763E-03	23.504	1.325E-03	23.679	7.205E-03	24.619	5.600E-03
	6	25.151	3.191E-03	25.870	6.743E-03	25.763	6.735E-03	25.629	3.629E-03	25.947	8.951E-03	25.528	5.725E-03	26.264	6.569E-03
	8	27.693	1.611E-03	27.568	5.885E-03	27.564	7.037E-03	27.010	6.345E-03	27.562	5.121E-03	27.643	3.478E-03	28.738	9.788E-03
M4	2	19.456	5.739E-03	19.662	2.007E-03	19.112	5.677E-03	19.767	5.629E-03	19.655	8.901E-03	19.410	8.401E-03	21.880	2.984E-03
	4	24.747	3.670E-03	24.749	6.960E-03	24.721	8.089E-03	24.602	3.957E-03	24.710	5.204E-03	24.956	2.109E-03	26.284	8.283E-03
	6	26.905	1.432E-03	26.565	9.955E-03	26.348	2.443E-03	26.068	8.874E-03	26.538	3.458E-03	26.470	4.586E-03	28.097	3.704E-03
	8	27.140	3.514E-03	27.161	6.335E-03	27.645	7.075E-03	27.049	2.880E-03	27.365	3.673E-03	27.345	4.103E-03	29.496	8.574E-03
M5	2	19.663	2.032E-03	19.632	8.636E-03	19.830	3.707E-03	19.784	6.801E-03	19.328	6.429E-03	19.517	7.000E-03	20.205	9.549E-03
	4	24.327	1.392E-03	24.252	8.829E-03	24.010	1.226E-03	24.459	4.570E-03	24.744	2.020E-03	24.295	9.182E-03	25.328	2.723E-03
	6	26.819	4.773E-03	26.906	6.321E-03	26.354	4.224E-03	26.066	9.712E-03	26.595	1.205E-03	26.808	1.429E-03	28.988	6.463E-03
	8	27.753	4.535E-03	27.473	1.993E-03	27.630	1.201E-03	27.783	2.196E-03	27.028	4.793E-03	27.126	5.627E-03	29.095	2.151E-03
M6	2	16.134	1.629E-03	16.211	2.258E-03	16.924	4.402E-03	16.142	5.497E-03	16.179	6.885E-03	16.978	2.878E-03	17.812	1.259E-03
	4	23.180	5.601E-03	23.060	7.456E-03	23.411	3.304E-03	23.253	5.801E-03	23.314	7.282E-03	23.281	9.382E-03	24.843	8.732E-03
	6	25.770	5.211E-03	25.903	4.970E-03	25.786	2.012E-03	25.277	9.380E-03	25.564	5.360E-03	25.142	7.188E-03	26.863	9.361E-03
	8	27.463	4.355E-03	27.491	5.340E-03	27.933	2.346E-03	27.944	8.938E-03	27.347	6.048E-03	27.985	7.104E-03	28.517	6.524E-03
M7	2	16.881	8.513E-03	16.738	2.467E-03	16.464	3.185E-03	16.108	4.967E-03	16.908	6.845E-03	16.567	7.951E-03	17.856	8.902E-03
	4	25.752	3.728E-03	25.456	4.500E-03	25.609	9.070E-03	25.079	1.109E-03	25.231	4.139E-03	25.766	2.919E-03	26.279	3.228E-03
	6	26.066	9.859E-03	26.704	9.148E-03	26.221	4.796E-03	26.684	3.847E-03	26.139	6.956E-03	26.579	3.714E-03	27.750	2.455E-03
	8	27.839	1.310E-03	27.431	7.825E-03	27.010	5.653E-03	27.722	4.842E-03	27.920	8.903E-03	27.360	3.586E-03	28.363	6.203E-03
M8	2	19.551	9.617E-03	19.690	2.887E-03	19.929	8.892E-03	19.949	5.924E-03	19.185	9.063E-03	19.368	3.091E-03	19.857	8.188E-03
	4	23.898	4.583E-03	23.223	1.979E-03	23.720	4.770E-03	23.812	7.948E-03	23.586	4.505E-03	23.749	7.421E-03	24.202	6.160E-03
	6	25.928	5.337E-03	25.531	3.288E-03	25.632	4.981E-03	25.034	5.608E-03	25.455	7.009E-03	25.273	6.643E-03	26.119	4.355E-03
	8	27.701	8.436E-03	27.934	1.717E-03	27.359	5.122E-03	27.309	6.183E-03	27.713	4.118E-03	27.840	8.713E-03	28.562	7.674E-03
Friedman Rank		4.53		4.06		4.41		4.84		4.53		4.44		1.19	
Final Rank		5		2		3		7		5		4		1	

Supplementary Table S3. The SSIM value of algorithms in mural image segmentation.

Image	nTH	LSHADE		IMODE		MGMSA		NGO		RAVWOA		PLO		OPBNGO	
		Mean	Std	Mean	Std	Mean	Std	Mean	Std	Mean	Std	Mean	Std	Mean	Std
M1	2	0.755	6.491E-03	0.759	5.136E-03	0.758	8.494E-05	0.757	9.259E-05	0.753	5.820E-06	0.756	4.351E-05	0.773	1.621E-07
	4	0.780	3.260E-03	0.786	3.075E-03	0.784	9.669E-03	0.788	6.367E-05	0.787	8.639E-04	0.781	4.293E-05	0.807	1.846E-07
	6	0.824	8.714E-03	0.829	7.906E-03	0.821	3.642E-03	0.829	4.318E-05	0.823	2.446E-04	0.820	6.093E-05	0.840	1.384E-07
	8	0.879	8.589E-03	0.872	7.699E-04	0.872	2.280E-03	0.871	3.837E-05	0.872	3.138E-04	0.879	9.803E-05	0.878	1.350E-07
M2	2	0.782	4.212E-03	0.788	1.473E-03	0.786	8.829E-03	0.784	2.487E-05	0.789	6.747E-04	0.782	5.505E-05	0.798	1.584E-07
	4	0.805	8.211E-03	0.806	7.253E-03	0.801	2.730E-03	0.804	7.385E-05	0.805	4.865E-04	0.804	2.822E-05	0.835	1.662E-07
	6	0.823	5.797E-03	0.821	6.394E-03	0.825	6.930E-03	0.826	9.654E-05	0.822	3.120E-04	0.823	5.183E-05	0.860	1.713E-07
	8	0.871	2.496E-03	0.877	2.251E-03	0.871	1.954E-03	0.877	1.656E-05	0.876	2.061E-04	0.877	4.751E-05	0.898	1.744E-07
M3	2	0.718	6.899E-03	0.714	5.649E-03	0.713	4.559E-03	0.719	2.979E-05	0.715	8.523E-04	0.714	4.617E-05	0.733	1.035E-07
	4	0.756	9.306E-03	0.756	7.849E-03	0.750	1.763E-03	0.753	3.148E-05	0.753	7.873E-04	0.751	7.857E-05	0.781	1.828E-07
	6	0.803	5.756E-03	0.806	1.629E-04	0.805	8.059E-03	0.805	4.347E-05	0.805	8.437E-04	0.800	2.328E-05	0.831	1.434E-07
	8	0.855	7.692E-03	0.850	3.156E-03	0.858	4.707E-03	0.853	1.801E-06	0.852	4.701E-04	0.857	8.628E-05	0.874	1.089E-07
M4	2	0.734	2.679E-03	0.731	6.389E-03	0.737	3.563E-03	0.733	5.162E-05	0.731	3.418E-04	0.733	3.963E-05	0.757	1.463E-07
	4	0.781	7.682E-03	0.786	6.618E-03	0.783	5.177E-03	0.783	7.046E-05	0.781	8.624E-05	0.784	5.096E-05	0.809	1.460E-07
	6	0.818	8.012E-03	0.816	4.326E-03	0.817	3.343E-03	0.811	2.230E-05	0.813	2.858E-04	0.814	5.495E-05	0.845	1.258E-07
	8	0.852	3.665E-03	0.850	3.610E-03	0.852	3.016E-04	0.853	7.811E-05	0.851	9.323E-07	0.853	7.479E-05	0.881	1.130E-07
M5	2	0.716	1.612E-03	0.713	6.167E-03	0.719	2.188E-03	0.720	8.103E-05	0.710	8.097E-05	0.711	4.575E-05	0.746	1.513E-07
	4	0.783	9.689E-03	0.787	2.064E-03	0.790	9.826E-03	0.788	3.930E-05	0.786	9.396E-04	0.786	9.157E-05	0.826	1.328E-07
	6	0.817	2.707E-03	0.819	1.864E-03	0.811	7.479E-03	0.816	7.466E-05	0.812	9.554E-04	0.813	2.566E-05	0.855	1.694E-07
	8	0.861	5.140E-03	0.863	3.012E-04	0.869	9.564E-03	0.869	8.431E-05	0.870	2.009E-04	0.860	7.854E-05	0.890	1.080E-07
M6	2	0.691	8.884E-03	0.694	8.958E-03	0.698	9.103E-03	0.697	9.221E-05	0.699	9.105E-04	0.692	9.040E-05	0.724	1.733E-07
	4	0.754	6.950E-04	0.756	1.519E-03	0.760	3.309E-03	0.756	9.810E-05	0.752	5.013E-04	0.756	9.149E-05	0.762	1.761E-07
	6	0.801	6.403E-04	0.804	8.035E-03	0.807	6.998E-03	0.806	1.813E-05	0.808	6.641E-04	0.807	6.852E-05	0.817	1.706E-07
	8	0.859	3.958E-03	0.860	9.797E-03	0.858	5.487E-05	0.856	2.386E-05	0.860	1.209E-04	0.857	5.109E-05	0.869	1.860E-07
M7	2	0.789	8.740E-03	0.784	2.089E-03	0.783	8.626E-03	0.782	4.747E-05	0.786	1.179E-04	0.782	7.183E-05	0.812	1.244E-07
	4	0.830	6.873E-03	0.828	1.955E-03	0.828	7.420E-03	0.822	9.800E-05	0.827	4.324E-04	0.821	9.886E-06	0.853	1.615E-07

	6	0.858	5.141E-03	0.852	2.338E-03	0.853	1.496E-03	0.853	6.601E-05	0.853	2.452E-04	0.858	3.049E-05	0.880	1.474E-07
	8	0.885	9.034E-03	0.889	3.393E-03	0.885	7.468E-03	0.889	7.998E-05	0.889	8.355E-04	0.890	5.093E-05	0.893	1.419E-07
M8	2	0.798	3.033E-03	0.796	3.295E-03	0.795	7.053E-04	0.795	9.033E-05	0.793	8.857E-05	0.799	5.402E-05	0.811	1.560E-07
	4	0.829	2.050E-03	0.825	6.831E-03	0.820	5.298E-03	0.823	7.260E-05	0.825	3.891E-04	0.824	2.869E-05	0.841	1.334E-07
	6	0.878	6.591E-03	0.880	9.876E-03	0.878	5.126E-03	0.873	2.907E-05	0.871	9.162E-04	0.877	4.089E-05	0.871	1.453E-07
	8	0.899	2.853E-03	0.891	8.195E-03	0.891	3.669E-03	0.898	7.450E-05	0.900	8.876E-04	0.895	9.374E-06	0.901	1.736E-07
Friedman Rank		4.19		4.13		4.59		4.34		4.72		4.81		1.22	
Final Rank		3		2		5		4		6		7		1	

Supplementary Table S4. The FSIM value of algorithms in mural image segmentation.

Image	nTH	LSHADE		IMODE		MGMSA		NGO		RAVWOA		PLO		OPBNGO	
		Mean	Std	Mean	Std	Mean	Std	Mean	Std	Mean	Std	Mean	Std	Mean	Std
M1	2	0.799	1.369E-10	0.795	3.699E-10	0.791	2.525E-10	0.800	2.787E-10	0.799	2.976E-10	0.791	7.131E-10	0.801	2.444E-10
	4	0.823	4.232E-10	0.828	7.578E-10	0.829	6.471E-10	0.826	7.630E-10	0.821	8.471E-10	0.829	9.340E-10	0.840	1.795E-10
	6	0.858	7.473E-10	0.856	5.644E-10	0.854	3.856E-10	0.857	8.094E-10	0.855	8.798E-10	0.859	9.977E-10	0.846	8.159E-10
	8	0.877	9.415E-10	0.880	3.588E-10	0.878	2.681E-10	0.876	6.599E-10	0.876	7.201E-10	0.879	1.537E-10	0.881	7.373E-10
M2	2	0.796	2.499E-10	0.798	3.154E-10	0.794	7.943E-10	0.798	3.114E-10	0.794	1.635E-10	0.797	7.194E-10	0.805	8.471E-10
	4	0.825	2.024E-10	0.828	4.110E-10	0.823	3.668E-10	0.827	6.007E-10	0.822	4.077E-10	0.828	6.739E-10	0.847	5.402E-10
	6	0.851	1.819E-10	0.852	4.235E-10	0.852	9.112E-10	0.853	5.731E-10	0.857	6.295E-10	0.852	2.506E-10	0.876	7.844E-10
	8	0.877	6.349E-10	0.878	5.186E-10	0.878	9.910E-10	0.875	2.711E-10	0.877	5.568E-10	0.872	1.573E-10	0.899	7.006E-10
M3	2	0.809	4.102E-10	0.802	7.075E-10	0.802	7.903E-10	0.809	8.048E-10	0.800	4.773E-10	0.809	1.595E-10	0.817	1.153E-10
	4	0.838	9.865E-10	0.839	8.119E-10	0.831	3.370E-10	0.838	6.018E-10	0.831	2.252E-10	0.838	2.507E-10	0.857	9.581E-10
	6	0.869	6.862E-10	0.866	7.746E-10	0.863	8.086E-10	0.869	5.164E-10	0.863	9.871E-10	0.861	7.764E-10	0.882	4.840E-10
	8	0.873	3.501E-10	0.879	4.340E-10	0.879	2.207E-10	0.873	3.799E-10	0.871	8.574E-10	0.873	9.697E-10	0.893	8.777E-10
M4	2	0.777	6.481E-10	0.774	2.571E-10	0.776	3.319E-10	0.775	3.706E-10	0.775	7.657E-10	0.780	3.244E-10	0.808	7.446E-10
	4	0.809	2.675E-10	0.804	6.989E-10	0.809	1.490E-10	0.806	7.201E-10	0.805	1.594E-10	0.808	6.730E-10	0.825	6.230E-10
	6	0.830	1.765E-10	0.835	8.649E-10	0.832	2.162E-10	0.838	3.353E-10	0.834	8.443E-10	0.835	5.687E-10	0.856	7.071E-10
	8	0.859	6.535E-10	0.855	6.348E-10	0.853	5.701E-10	0.853	6.601E-10	0.858	7.130E-10	0.858	7.607E-10	0.892	3.794E-10
M5	2	0.783	5.426E-10	0.790	3.824E-10	0.788	8.168E-10	0.780	2.533E-10	0.783	1.722E-10	0.780	1.374E-10	0.795	5.232E-10
	4	0.811	2.669E-10	0.815	2.128E-10	0.812	1.302E-10	0.820	3.255E-10	0.814	6.819E-10	0.810	3.220E-10	0.831	5.088E-10
	6	0.836	5.925E-10	0.838	6.920E-10	0.834	5.066E-10	0.840	5.679E-10	0.835	4.682E-10	0.837	6.665E-10	0.880	8.167E-10
	8	0.856	5.098E-10	0.850	1.471E-10	0.851	7.486E-10	0.855	4.516E-10	0.856	3.550E-10	0.859	2.183E-10	0.895	8.323E-10
M6	2	0.754	4.311E-10	0.755	2.023E-10	0.757	2.196E-10	0.755	8.818E-10	0.752	8.866E-10	0.753	1.346E-10	0.780	2.156E-10
	4	0.795	9.290E-10	0.798	8.336E-10	0.790	9.380E-10	0.792	3.469E-10	0.795	9.763E-10	0.793	8.754E-10	0.817	3.710E-10
	6	0.823	2.849E-10	0.821	4.874E-10	0.821	8.160E-10	0.827	7.277E-10	0.824	6.571E-10	0.825	8.643E-10	0.842	8.907E-10
	8	0.856	9.860E-10	0.852	4.744E-10	0.855	5.517E-10	0.851	6.519E-10	0.857	7.350E-10	0.851	8.042E-10	0.886	1.727E-10
M7	2	0.813	3.598E-10	0.810	8.399E-10	0.813	5.921E-10	0.818	6.427E-10	0.814	2.563E-10	0.817	2.983E-10	0.827	9.843E-10
	4	0.839	3.231E-10	0.835	8.150E-10	0.837	3.087E-10	0.830	1.124E-10	0.833	5.147E-10	0.831	6.781E-10	0.847	6.809E-10
	6	0.863	4.014E-10	0.870	6.182E-10	0.869	7.214E-10	0.869	7.381E-10	0.868	9.885E-10	0.865	9.066E-10	0.871	2.569E-10
	8	0.872	3.330E-10	0.877	7.858E-10	0.878	5.386E-10	0.878	3.712E-10	0.877	9.733E-10	0.871	1.252E-10	0.898	8.869E-10
M8	2	0.822	9.841E-10	0.826	5.180E-10	0.822	1.489E-10	0.825	9.974E-10	0.824	9.000E-10	0.830	6.551E-10	0.828	3.783E-10
	4	0.848	5.057E-10	0.847	9.098E-10	0.843	4.445E-10	0.841	1.018E-10	0.843	2.889E-10	0.849	3.372E-10	0.873	6.997E-10
	6	0.864	6.456E-10	0.870	7.203E-10	0.869	3.641E-10	0.860	6.762E-10	0.860	9.239E-10	0.862	1.746E-10	0.890	8.320E-10
	8	0.894	3.182E-10	0.890	4.102E-10	0.891	6.346E-10	0.890	8.190E-10	0.895	3.728E-10	0.893	7.263E-10	0.905	1.483E-10
Friedman Rank		4.34		4.00		4.84		4.34		4.97		4.28		1.22	
Final Rank		4		2		6		4		7		3		1	

Supplementary Table S5. The runtime of algorithms in mural image segmentation.

Image	nTH	LSHADE	IMODE	MGMSA	NGO	RAVWOA	PLO	OPBNGO
M1	2	22.246	25.795	27.338	33.360	35.290	22.638	18.607
	4	29.795	27.131	25.576	32.865	38.343	24.036	17.951
	6	27.223	26.309	25.394	35.284	38.103	20.391	19.887
	8	26.073	23.234	31.312	34.863	35.487	22.262	20.461
M2	2	22.731	29.245	34.994	36.558	33.527	22.628	17.655
	4	24.774	21.245	28.257	34.921	34.346	21.695	15.753
	6	22.178	25.727	34.433	32.111	34.531	23.422	19.515
	8	26.589	25.469	31.368	30.126	33.648	20.456	15.289

M3	2	22.902	24.860	31.509	35.497	38.439	23.487	16.596
	4	21.870	24.139	29.661	31.239	37.125	22.397	20.700
	6	22.272	25.860	30.158	32.786	38.885	21.236	17.676
	8	23.259	27.190	33.644	36.582	34.452	23.210	17.656
M4	2	28.858	27.625	28.478	30.084	34.126	22.024	21.374
	4	21.687	23.066	30.074	33.331	34.631	21.085	21.432
	6	27.338	23.013	26.432	33.137	34.885	22.635	18.823
	8	21.539	20.466	32.098	35.553	37.036	22.109	17.693
M5	2	27.051	26.874	33.837	32.410	37.332	22.998	15.447
	4	21.156	24.370	33.087	36.324	37.619	24.490	15.044
	6	27.204	21.496	31.242	36.662	36.245	24.990	18.766
	8	20.162	28.583	26.688	36.515	34.655	23.567	21.327
M6	2	22.156	21.946	28.873	32.814	36.242	24.470	15.007
	4	27.657	21.651	25.626	33.669	37.880	20.017	19.296
	6	22.251	22.412	34.230	31.968	34.063	21.502	21.058
	8	27.238	26.874	31.548	32.546	37.574	22.039	18.613
M7	2	21.010	25.834	25.987	35.029	36.410	23.831	15.306
	4	29.874	21.031	34.177	34.068	34.794	20.810	16.437
	6	29.770	21.483	32.485	31.623	35.415	20.449	16.103
	8	22.189	25.675	28.008	34.989	33.067	23.508	16.860
M8	2	25.334	24.984	26.049	35.902	37.443	23.440	20.289
	4	25.261	25.928	29.747	36.550	36.099	24.613	18.110
	6	25.433	26.582	28.924	34.232	36.534	20.709	19.578
	8	21.515	28.109	29.030	33.060	35.941	22.732	20.371
Mean Rank		3.28	3.44	5.00	6.03	6.72	2.47	1.06
Final Rank		3	4	5	6	7	2	1
